# Melanoma-Derived Exosomes Induce PD-1 Overexpression and Tumor Progression via Mesenchymal Stem Cell Oncogenic Reprogramming

**DOI:** 10.3389/fimmu.2019.02459

**Published:** 2019-10-18

**Authors:** Edina Gyukity-Sebestyén, Mária Harmati, Gabriella Dobra, István B. Németh, Johanna Mihály, Ágnes Zvara, Éva Hunyadi-Gulyás, Róbert Katona, István Nagy, Péter Horváth, Árpád Bálind, Ábel Szkalisity, Mária Kovács, Tibor Pankotai, Barbara Borsos, Miklós Erdélyi, Zsolt Szegletes, Zoltán J. Veréb, Edit I. Buzás, Lajos Kemény, Tamás Bíró, Krisztina Buzás

**Affiliations:** ^1^Laboratory of Microscopic Image Analysis and Machine Learning, Institute of Biochemistry, Biological Research Centre of Hungarian Academy of Sciences, Szeged, Hungary; ^2^Doctoral School of Interdisciplinary Sciences, Faculty of Medicine, University of Szeged, Szeged, Hungary; ^3^Department of Dermatology and Allergology, University of Szeged, Szeged, Hungary; ^4^Department of Immunology, Faculty of Medicine, University of Debrecen, Debrecen, Hungary; ^5^Laboratory of Functional Genomics, Institute of Genetics, Biological Research Centre of Hungarian Academy of Sciences, Szeged, Hungary; ^6^Laboratory of Proteomics Research, Institute of Biochemistry, Biological Research Centre of Hungarian Academy of Sciences, Szeged, Hungary; ^7^Artificial Chromosome and Stem Cell Research Laboratory, Institute of Genetics, Biological Research Centre of Hungarian Academy of Sciences, Szeged, Hungary; ^8^Sequencing Platform, Institute of Biochemistry, Biological Research Centre of Hungarian Academy of Sciences, Szeged, Hungary; ^9^Department of Pharmacology and Pharmacotherapy, Faculty of Medicine, University of Szeged, Szeged, Hungary; ^10^Department of Biochemistry and Molecular Biology, Faculty of Science and Informatics, University of Szeged, Szeged, Hungary; ^11^Advanced Optical Imaging Group, Department of Optics and Quantum Electronics, Faculty of Science and Informatics, University of Szeged, Szeged, Hungary; ^12^Atomic Force Microscope Laboratory, Institute of Biophysics, Biological Research Centre of Hungarian Academy of Sciences, Szeged, Hungary; ^13^MTA-SE Immuno-proteogenomics Extracellular Vesicle Research Group, Department of Genetics, Cell- and Immunobiology, Faculty of Medicine, Semmelweis University, Budapest, Hungary; ^14^Department of Oral Biology and Experimental Dental Research, Faculty of Dentistry, University of Szeged, Szeged, Hungary

**Keywords:** PD-1, exosome, melanoma/tumor progression, stem cell, reprogramming, signalization pattern, metastasis

## Abstract

Recently, it has been described that programmed cell death protein 1 (PD-1) overexpressing melanoma cells are highly aggressive. However, until now it has not been defined which factors lead to the generation of PD-1 overexpressing subpopulations. Here, we present that melanoma-derived exosomes, conveying oncogenic molecular reprogramming, induce the formation of a melanoma-like, PD-1 overexpressing cell population (mMSC^PD-1+^) from naïve mesenchymal stem cells (MSCs). Exosomes and mMSC^PD-1+^ cells induce tumor progression and expression of oncogenic factors *in vivo*. Finally, we revealed a characteristic, tumorigenic signaling network combining the upregulated molecules (e.g., PD-1, MET, RAF1, BCL2, MTOR) and their upstream exosomal regulating proteins and miRNAs. Our study highlights the complexity of exosomal communication during tumor progression and contributes to the detailed understanding of metastatic processes.

## Introduction

Heterogeneous tumor tissue is comprised of a wide variety of collocated cells. Their spatiotemporal co-existence facilitates direct communication between them. Cancer cells contribute to tumor niche formation not only by soluble factor production and receptor-ligand interactions (1), but also by releasing vesicles whose molecular contents add up to a complex information package. Previous studies demonstrated that cultured human tumor cells release extracellular vesicles such as exosomes of 20–120 nm diameters (2). Among others, exosomes carry structural and signaling proteins, MHC molecules, cell surface molecules typically associated with apoptosis, and mRNAs/miRNAs with multiple functions (3). Therefore, these exosomal-molecular-patterns, as unique entities of the complex intercellular communication, are not independent of the quality or state of the mother cell (4, 5). Exosomes have been recognized long ago, but their identification, characterization, and isolation are still under intense investigation. Further, whereas the definition of exosomes is based on the pathway of biogenesis, oncosomes form a functional class of extracellular vesicles. Indeed, oncosomes are suggested being capable of carrying and conveying tumor-related information (6) and might have a significant role in formation of tumor microenvironment (7).

Mesenchymal stem cells (MSCs) were first described as stromal cells of the bone marrow with multipotent differentiation potential and characteristic immunomodulatory effects (8). In relation to their immunological and differentiation properties, there is a debate about the role of MSCs in tumor progression (9). Indeed, the cellular fate could depend on the cancer type and also on the status of the affected MSCs. Activated MSCs can secrete pro-angiogenic soluble factors and are able to differentiate to vessel wall associated cells (10) or to cancer associated fibroblasts (CAFs) (11). Furthermore, Baglio et al. showed that tumor secreted extracellular vesicles promote osteosarcoma progression via TGFβ signaling induced IL-6 production by MSCs (12). Moreover, Peinado et al. demonstrated that exosomes from highly metastatic melanomas increase the metastatic behavior of primary tumors by permanently educating bone marrow progenitors through the receptor tyrosine kinase MET (13).

Malignant melanoma is a highly invasive, metastatic cancer with poor prognosis and survival rate. Since melanoma has been long recognized as a highly immunogenic tumor, therapeutic approaches target different immunological mechanisms to treat patients with this aggressive skin cancer. In the past decade, besides IL-2 and CTLA-4 therapies, the PD-1:PD-L1 blockade proved to be an effective treatment in metastatic melanoma (14). Programmed cell death protein 1 (PD-1), mainly expressed by immune cells such as activated T cells, dendritic cells and macrophages is a cell surface receptor with a central role in modulation of T cell responses. PD-1 binding to its ligand, PD-L1, can result in apoptosis and anergy of immune cells.

Notably, PD-1 expression is not a default property of melanoma cells. Of further importance, recently Kleffel et al. demonstrated that PD-1 overexpressing melanoma cell subpopulations are especially aggressive, and that melanoma PD-1:PD-L1 interactions modulate mTOR signaling, whereby they interfere with programmed cell death (15). This work is remarkably instrumental with respect to the consequences of PD-1 overexpression and experimental inhibition of PD-1 functions (Pdcd1-shRNA or anti-PD-1 antibody). However, it does not define the factors the activation of which lead to the generation of PD-1 overexpressing subpopulations. This question, though, is an especially intriguing one, if one considers that one of the most effective immunotherapies available today is based on the blockade of the aforementioned PD-1:PD-L1 interaction (16).

Based on the above, in the current study, we aimed at specifically and systematically addressing the following questions:

1. Can we unambiguously define the cellular and molecular signs of melanoma-derived exosome-induced, intercellular communication-mediated malignant transformation of MSC cultures?
2. Can we detect the melanoma-derived exosome-induced tumor progression *in vivo*?
3. What alteration can be detected in the expression of PD-1, a melanoma progression marker and therapeutic target, upon exposure to melanoma-derived exosomes?

Here, we present the first piece of evidence that melanoma exosomes, by conveying a complex oncogenic molecular reprogramming, induce the formation of a PD-1 overexpressing cell population (melanoma-like MSC^PD-1+^; mMSC^PD-1+^) from naïve MSCs. These mMSC^PD-1+^ cells represent a new entity with melanoma-like gene expression profile and phenotypic properties. Further, exosomes and exosome-activated mMSC^PD-1+^ cells induce rapid tumor progression *in vivo*, due to their strong expression of oncogenic dominance factors and decreased susceptibility to programmed cell death.

## Materials and Methods

### Cell Culture and Cell Line

B16F1 mouse melanoma cell line was obtained from ECACC and cultured in Dulbecco's modified Eagle's medium (DMEM, Lonza, Basel, Switzerland) containing 10% fetal bovine serum (FBS) (Euroclone, Milano, Italy), 1% sodium pyruvate, 1% MEM non-essential amino acids, 1% MEM vitamin solution, 2 mM L-glutamine, and 1% Penicillin-Streptomycin-Amphotericin B Mixture (all from Lonza).

Murine MSCs were isolated from C57BL/6N (Charles River Laboratories, Sulzfeld) 8 week-old male mice (17). Abdominal inguinal fat pads were excised, rinsed with RPMI 1640 medium, transferred to sterile tissue culture dishes and mechanically dissociated. The dissociated tissue was resuspended in RPMI 1640 containing 100 μg/ml collagenase (Sigma-Aldrich, St. Louis, MO) and incubated at 37°C for 50 min. Collagenase was neutralized with a growth medium containing 10% FBS. After centrifugation at 470 g for 15 min, cell pellets were resuspended and washed in the culture medium. After the centrifugation, cell pellets were resuspended in complete MesenCult medium (Stemcell Technologies, Vancouver, British Columbia, Canada) and filtered through a 100 μm cell strainer (BD Biosciences, Franklin Lakes, NJ) to tissue culture dishes, and cultured for 48 h at 37°C in 5% CO_2_ and 90% humidity. Unattached cells and debris were then removed and fresh medium was added to the adherent cells. The cells were cultured to 80% confluence before being released with trypsin–EDTA and sub-cultured.

Purity of MSC cultures were checked by flow cytometry analysis using the Mouse Multipotent Mesenchymal Stromal Cell Marker Antibody Panel (R&D Systems, Minneapolis, MN) according to the manufacturer's instructions.

### Isolation and Characterization of the Melanoma-Derived Exosomes

#### Exosome Isolation by Filtration and Differential Centrifugation

Exosomes were isolated by adapting the protocol of Peinado et al. (13) from melanoma cell culture supernatant. B16F1 supernatants were harvested, supplemented by complete protease inhibitor cocktail (Roche, Mannheim, Germany) and centrifuged at 780 g for 5 min at 4°C to remove intact cells. Then, the supernatants were centrifuged at 3,900 g for 15 min at 4°C and filtered through a 0.2 μm membrane (Millipore, Billerica, MA) to remove larger cell fragments and microvesicles. Exosomes were pelleted by ultracentrifugation at 150,000 g (T-1270 rotor at 40,500 rpm) for 1 h at 4°C. The pellet was washed twice and resuspended in DPBS and stored at −80°C.

The concentration of exosomal proteins was determined using a Pierce BCA Protein assay kit (Thermo Fisher Scientific, Waltham, MA) and a Benchmark Microplate Reader (Bio-Rad, Hercules, CA) according to the manufacturer's instructions.

#### Determination of Shape and Size of the Isolated Vesicles

##### Scanning electron microscopy (SEM)

Five microliters of isolated exosomes in DPBS were spotted on glass coverslips coated with 0.01% Poly-L-Lysine (Sigma-Aldrich) and incubated overnight at 4°C. Using sterile forceps, coverslips were transferred into a plastic plate. Exosomes were fixed by gently adding 2% paraformaldehyde buffer pH 7.2 diluted in DPBS for 30 min. The coverslips were washed twice with DPBS and dehydrated with a graded ethanol series (40, 60, 80, and 100% ethanol, each for 5 min). The samples were dried with a critical point dryer (QUORUM K850, Quorum Technologies Ltd, Laughton, UK) and the coverslips were mounted onto a microscope stub at a time using carbon tape, followed by 7 nm gold coating (QUORUM Q150, Quorum Technologies Ltd) and observed under a field-emission scanning electron microscope (JEOL JSM-7100F/LV).

##### Atomic force microscopy (AFM)

Exosomes were let to adsorb to the freshly cleaved muscovite mica (SPI-ChemTM Mica Sheets, Structure Probe, Inc., West Chester, PA) surface directly from DPBS. All AFM measurements were carried out with an Asylum MFP-3D head and Molecular Force Probe Controller (Asylum Research, Santa Barbara, CA), using Asylum Research MFP-3D program (version 15.09.112) written in Igor Pro software (version 6.37, Wavemetrics, Lake Oswego, OR). Image procession and data calculation were made using the same software. Silicon rectangular cantilevers (OMLC-AC240TS, Olympus Optical Co., Ltd., Tokyo, Japan) with the typical spring constant of 2 N/m were used in dry condition and silicon nitride rectangular cantilevers with “V” shaped tips (Bio-Lever BL-RC150VB, Olympus Optical Co. Ltd.) with the typical spring constant of 0.03 N/m in buffer solution. Typically, 512 × 512 points was taken at 1 line/s scan rate. The measurements presented here are amplitude images and height profile.

#### Detection of Exosomal Markers by Western Blotting

Protein samples were resuspended in 4x sample buffer (NuPAGE LDS Sample Buffer (4X), NuPAGE Sample Reducing Agent (10X), Thermo Fisher Scientific), boiled at 96°C for 10 min, and immediately cooled on ice. Electrophoresis of the proteins was performed using 4–12% Bis-Tris Protein Gels (NuPAGE Novex, Thermo Fisher Scientific), ProSieve Color Protein Markers (Lonza), and electrophoresis buffer (NuPAGE MOPS SDS Running Buffer, Thermo Fisher Scientific) at 200 V and 0.03 A for 40 min.

The proteins of electrophoresed gels were transferred to Immobilon transfer membrane (Millipore, Darmstadt, Germany) using transfer buffer [NuPAGE Transfer Buffer (20X)] at 30 V and 170–110 mA for 60 min. Membranes were blocked in TBST buffer (25 mM Tris-HCl, 150 mM NaCl, 0.05% Tween-20, pH 7.2) containing 5% non-fat milk for 60 min at room temperature (RT). After blocking, membranes were incubated with each primary antibody in TBST buffer containing 1% non-fat milk overnight at 4°C. Membranes were washed three times for 10 min with TBST buffer and incubated for 60 min at room temperature with secondary antibody in TBST buffer containing 1% non-fat milk. Membranes were washed three times for 10 min with TBST buffer.

For exosomal marker identification western blot analyses were performed with an anti-CD63 polyclonal antibody (1:250 dilution; Biorbyt, Cambridge, UK) anti-CD9 monoclonal antibody (clone EPR2949, 1:500 dilution, LifeSpan Biosciences, Seattle, WA), anti-CD81 monoclonal antibody (clone: EAT2, 1:1,000 dilution, LifeSpan Biosciences, Seattle, WA), and anti-HSP70 monoclonal antibody (clone: C92F3A-5, 1:8,000 dilution; Enzo Life Sciences, Farmingdale, NY), anti-rabbit IgG HRP-Conjugated antibody (1:1,000 dilution, R&D Systems), anti-Hamster IgG HRP-Conjugated antibody (1:30,000 dilution, Thermo Fisher Scientific). Bound antibodies were visualized by chemiluminescence using an ECL Plus Western Blotting detection system (Advansta, Menlo Park, CA). Immunoreactive signals were detected by using LI-COR ODYSSEY Fc (Dual-mode imaging system) imager followed by analysis with Odyssey v1.2, Image Studio Lite v5.2.

#### Identification of Exosomal Proteins by Mass Spectrometry

Exosomal proteins (24 μg) were separated in 4–12% Bis-Tris Protein Gels and stained with Coomassie blue (0.1% Coomassie Brilliant Blue R-250, 50% methanol and 10% acetic acid).

Each lane was cut to 12–12 equal bands and subjected to in-gel digestion. Gel bands were diced to smaller pieces, and the SDS and CBB dyes were washed out with 3 × 50 μl 25 mM ammonium-bicarbonate (ABC)/50% Acetonitrile (AcN). After reduction with DTT (1,4-dithiothreitol, Sigma-Aldrich; 20 μl, 10 mM DTT in 25 mM ABC) at 56°C for 30 min, and alkylation with IAM (iodoacetamide, Sigma- Aldrich; 20 μl, 55 mM IAM in 25 mM ABC) at RT in the dark for 30 min, the gel samples were dried in a vacuum centrifuge and after that rehydrated in 20 μl of trypsin (Sequencing Grade Modified Trypsin, Promega, Fitchburg, WI; 5 ng/μl in 25 mM ABC) and incubated at 37°C. The digestion was stopped after 4 h by lowering the pH of the buffer below 3, by adding 2 μl of 10% formic acid. Tryptic peptides were extracted from the gel with 3 × 50 μl of 2% formic acid in 50% AcN and dried. Prior mass spectrometric analysis, all samples were redissolved in 50–50 μl of 0.1% formic acid (FA).

Samples were analyzed on an LTQ-Orbitrap Elite (Thermo Fisher Scientific) mass spectrometer on-line coupled with a nanoHPLC (nanoAcquity, Waters, Milford, MA) system. 5–5 μl of the in-gel digests were loaded (for 3 min at 8 μl/min flow, using 0.1% FA in 3% Acetonitrile−97% water) onto a reversed phase trap column (Waters, Symmetry C18, 0.180 × 20 mm) and separated on a C18 reversed phase (Waters, Milford, MA, BEH300C18 1.7 μm) nanocolumn (0.075 × 200 mm). The flow rate was 330 nl/min and a linear gradient was used from 3 to 40% B in 37 min (solvent A was 0.1% FA in water and solvent B was 0.1% FA in Acetonitrile).

The high voltage (1.2 kV) was applied through liquid junction between the chromatographic column and the non-coated silica nanospray emitter (NewObjective, Woburn, MA, 10 μm tip ID). The mass spectrometer operated in data-dependent mode: the survey mass spectra were detected in the orbitrap with high resolution (R = 60 k @ m/z: 400, mass range m/z: 380–1,400) and the most abundant multiply charged 20 peaks were selected for ion-trap fragmentation (NCE: 35%; activation q: 0.25; activation time: 10 ms; minimum signal intensity: 5,000 counts). The MSMS spectra were detected in the ion trap. Dynamic exclusion was used, the precursors were excluded for 15 s after the first fragmentation event.

Data analysis: searchable peaklists (mgf format) were extracted using ProteomeDiscoverer (ver:1.4 Thermo Fisher Scientific) and subjected to database search on our in-house ProteinProspector (ver: 5.14.1) search engine using the following parameters: parent ion tolerant: 5 ppm; fragment ion tolerant: 0.6 Da; Cys carbamidomethylation was set as constant and Met oxidation, cyclisation of peptide N-terminal Glu to pyroglutamic acid, protein N terminal acetylation were set as variable modifications. Only fully tryptic peptides were considered with maximum of 2 missed cleavage sites. The *Mus musculus* and *Bos taurus* protein sequences of the Uniprot (UniProtKB.06.11.2014) database completed with human keratins and pig trypsin, altogether 106,330 protein sequences were searched. For the false discovery rate (FDR) estimation, the searches were performed on the database concatenated with the randomized sequences. Protein identification was accepted if the ProteinProspector expectation value was <0.01 and the protein was identified with at least 2 unique peptides (expectation value <0.05 and score higher than 15). FDR values were <1% in all cases.

For functional validation, the resulted protein list was analyzed by the “Core Analysis” function included in Ingenuity Pathway Analysis (IPA, Quiagen Bioinformatics) software.

#### Isolation and Detection of Exosomal miRNAs

miRNA sequencing was performed using SOLiD Total RNA-Seq lit for Small RNA Libraries (Applied Biosystems now part of Thermo Fisher Scientific) according to the manufacturer's instructions. Purification was performed on 10% TBE-Urea gels stained with Sybr Gold nucleic acid gel stain (both from Invitrogen now part of Thermo Fisher Scientific). Final purification was performed using PureLink PCR Micro Kit (Invitrogen). Final libraries were quality checked using High Sense DNA kit on Bioanalyzer (all from Agilent, Santa Clara, CA). Concentration of each library was determined using the SOLID Library TaqMan Quantitation Kit (Life Technologies now part of Thermo Fisher Scientific). Each library was clonally amplified on SOLiD P1 DNA Beads by emulsion PCR (ePCR). Emulsions were broken with butanol, and ePCR beads enriched for template-positive beads by hybridization with magnetic enrichment beads. Template-enriched beads were extended at the 3′ end in the presence of terminal transferase and 3′ bead linker. Beads with the clonally amplified DNA were deposited onto SOLiD sequencing slide and sequenced on SOLiD 5500 Instrument using the 50-base sequencing chemistry.

Bioinformatic Analysis Raw data quality assessment, read trimming read mapping and miRNA expression profiling were carried out in CLC Genomics Workbench tool version 8.0.2 (CLC Bio now part of Qiagen, Venlo, Netherlands) using annotated *Mus musculus* miRNA sequences according to the miRBase release 21 as a mapping reference.

### *In vitro* Experiments

#### Cell Cultures

6 × 10^4^ cell/ml passage 2 MSCs were plated in cell culture dishes (1.5 × 10^4^/cm^2^). After 24 h incubation, MSC cultures were exposed to B16F1-derived exosomes (40 μg/ml exosomal proteins; 1.5 × 10^11^ exosomes) at every 24 h. Samples were exposed to exosomes for 24, 48, 72, and 96 h and then harvested in method-competent buffers.

#### Visualization of Labeled Exosome Internalization in MSCs

To examine the uptake of exosomes by MSCs, cells were plated to black 24-well Visiplates (1 × 10^4^ cells/well) and incubated for 24 h. The exosomes were labeled with Dil dye (1,1′-dioctadecyl-3,3,3′,3′-tetramethylindocarbocyanine perchlorate, PromoKine, Heidelberg, Germany) and the MSC cultures were labeled with DiO dye (3,3′-dioctadecyloxacarbo-cyanine perchlorate, PromoKine) according to the manufacturer's instructions. Dil-labeled exosomes were washed in DPBS by ultracentrifugation (at 150,000 g for 1 h at 4°C). Forty micrograms per milliliter DiL-labeled exosomes were added to DiO-labeled MSC cultures and the exosome uptake was followed for 24 h in the Celldiscoverer 7 automated live cell imaging system (Zeiss, Oberkochen, Germany). After 24 h, the cells were fixed with 4% paraformaldehyde solution and a nucleus staining was performed using DAPI (Life Technologies now part of Thermo Fisher Scientific). Then, 5 image z-stacks were acquired for both channels by Operetta High Content Screening System (Perkin Elmer, Waltham, MA). The stacks were maximum intensity projected and then analyzed automatically using a customized version of CellProfiler (18). Nuclei were detected with Otsu-adaptive threshold combined with diameter based filtering, then cytoplasms were identified with propagation method seeded from the nuclei and using the exosome channel. Exosomes were located with a customized version of A-trous wavelet transform based spot detection (19). Several wavelet levels were used to ensure the detection of exosomes with various size and then the overlaps were removed based on circularity measures. Finally, the exosome numbers per cell were identified using MATLAB programming, the diagrams were created in Microsoft Excel.

#### Cell Proliferation

After 72 h incubation, exosome-exposed and control MSC cultures were dissociated with trypsin from the culture surface. Cells were washed in medium and counted in a Bürker chamber and a cell counter (Bio-Rad, TC10 Automated Cell Counter).

#### Detection of Apoptosis

Exosome-exposed MSCs and control cells were treated with 100 ng/ml mouse TNFα (R&D Systems). After 24 h incubation, cell death was determined by the Annexin V Apoptosis Detection Kit with PI (Biolegend, San Diego, CA) according to the manufacturer's recommendations. Samples were measured by FACS Calibur flow cytometer (BD Biosciences), data were analyzed by Flowing Software (Cell Imaging Core, Turku Center for Biotechnology, Finland) where percent of positive cells was determined by relative fluorescence intensity and the results were expressed as mean of percentage of positive cells (%) ±SD. Cells that are annexin-V/PI double positive show the sign of late apoptosis, while cells that are annexin-V positive and PI negative indicate early apoptosis. Annexin-V negative and PI positive cells are necrotic, viable cells are both annexin-V, and propidium iodide (PI) negative.

#### RNA Preparation, Melan-A, and Mitf Quantitative Real-Time PCR (QRT-PCR)

Total RNA of biological samples was purified using the Quick-RNA MiniPrep isolation kit of #R1054S (Zymo Research Irvine, CA). All the preparation steps were carried out according the manufacturer's instructions. RNA samples were stored at −80°C in the presence 30 U of Prime RNAse inhibitor (Fermentas, part of Thermo Fisher Scientific) for further analysis. The quantity of isolated RNA samples was checked by spectrophotometry (NanoDrop 3.1.0, Rockland, DE).

QRT-PCR was performed on a RotorGene 3000 instrument (Corbett Research, Sydney, Australia) with gene-specific primers and SybrGreen protocol to monitor gene expression. One microgram of total RNA was reverse transcribed with random primers using the High-Capacity cDNA Archive Kit (Applied Biosystems) according to the manufacturer's instructions in final volume of 30 μl. The temperature profile of the reverse transcription was the following: 10 min at room temperature, 2 h at 37°C, 5 min on ice, and finally 10 min at 75°C for enzyme inactivation. These steps were carried out in a Thermal Cycler machine (MJ Research, Marshall Scientific, Hampton, NH). After dilution with 30 μl of water, 1 μl of the diluted reaction mix was used as template in the QRT-PCR. Reactions were done with FastStart SYBR Green Master mix (Roche) according to the manufacturer's instructions at a final primer concentration of 250 nM under the following conditions: 15 min at 95°C, 40 cycles of 95°C for 15 s, 60°C for 25 s, and 72°C for 25 s. The fluorescence intensity of SybrGreen dye was detected after each amplification step. Melting temperature analysis was done after each reaction to check the quality of the products. Primers were designed using the online Roche Universal Probe Library Assay Design Center. The quality of the primers was verified by MS analysis provided by Bioneer (Daejeon, Republic of Korea). Relative expression ratios were calculated as normalized ratios to MmRpl27 (*Mus musculus* ribosomal protein L27) gene. Non-template control sample was used for each PCR run to check the primer-dimer formation. The final relative gene expression ratios were calculated as delta-delta Ct values. Information about the genes and the primers is collected in Table S1.

#### RNA Isolation, Reverse Transcription, and TaqMan Panel

RNAs were isolated from MSC cells with Qiagen RNeasy mini kit (Qiagen) based on the manufacturer's instruction. RNA concentrations were measured by NanoDrop spectrophotometer (NanoDrop). cDNAs were reverse transcribed with TaqMan Reverse Transcription Reagents (Thermo Fisher Scientific) following the manufacturer's instruction. For the TaqMan QRT-PCR panel, cDNA mixture of three parallel samples was used in case of each condition: control, 6, 24, and 72 h exosome treatment applied on MSC cells derived from four mice. Eighty nanogram cDNA and TaqMan Gene Expression Master Mix (Thermo Fisher Scientific) were used for the qPCR experiment. Expression of the examined 44 genes was calculated by ΔΔCt method and normalized to the average Ct values of 4 internal controls (PPIA, 18S RNA, ACTB, and GAPDH).

#### PD-1 Detection

##### PD-1 detection by Western blotting

The cells were washed three times in PBS, then were lysed in TENT Buffer (50 mM Tris-HCl, 2 mM EDTA, 150 mM NaCl, 1% TritonX-100, completed with 1x protease inhibitor cocktail (Roche). Protein samples were separated and blotted as described above.

For PD-1 protein level detection, anti-PD-1 (clone: RMO1-14, Biolegend) antibody was used in 1:1,000 dilution. Anti-rat horseradish peroxidase (HRP)-conjugated secondary antibody (R&D Systems) was used in 1:500 dilution.

##### PD-1 detection by Immunofluorescence microscopy

MSC cultures were fixed with 4% paraformaldehyde for 10 min, and blocked in PBS buffer containing 5% BSA (Sigma-Aldrich) for 60 min at room temperature. After blocking, cells were incubated with primary antibody in DPBS buffer containing 1.2% BSA overnight at 4°C. As primary antibodies, 1:200 anti-PD-1 (clone: RMO1-14, Biolegend,) and 1:200 anti-MLANA (Byorbit) were used. After incubation, cells were washed three times in PBS, and incubated for 1 h at room temperature with 1:100 Alexa Fluor 647-conjugated anti-rat antibody (Jackson ImmunoResearch Laboratories, Baltimore, PA) or 1:500 Alexa Fluor 555-conjugated anti-rabbit antibody (Thermo Fisher Scientific). Tubulin was stained with 1:500 Alexa Fluor 488-conjugated anti-tubulin-α antibody (clone: 10D8, Biolegend). Nucleus staining was performed using DAPI (Life Technologies). Slides were washed three times with DPBS between each step. Images were obtained at 60x magnification using an Olympus confocal laser scanning microscope.

##### PD-1 detection by STORM super-resolution imaging

All dSTORM super-resolution experiments were performed on a custom-made inverted microscope based on a Nikon Eclipse Ti-E frame. After being conditioned (through spatial filtering via fiber coupling and beam expansion) the applied laser beams were focused into the back focal plane of the microscope objective (Nikon CFI Apo 100x, NA = 1.49), which produced a collimated beam on the sample. The angle of illumination was set via a tilting mirror mounted into a motorized gimbal holder and placed into the conjugate plane of the sample. All the dSTORM images were captured under EPI illumination at an excitation wavelength of 647 nm (Nikon: 647 nm, 300 mW). The laser power, controlled via an acousto-optic tunable filter (AOTF), was set to 4 kW/cm^2^ on the sample plane. An additional laser (Nichia, Anan, Tokushima, Japan, 405 nm, 60 mW) was used for both reactivation and reference measurements. Images were captured by an Andor iXon3 897 BV EMCCD digital camera (512 × 512 pixels with pixel size of 16 μm). The size of the illuminated region of the sample was matched to the size of the detector, which determined the field of view (FOV = 80 × 80 μm^2^). Frame stacks for dSTORM super-resolution imaging were typically captured at a reduced image size (crop mode), when only the central 128 × 128 pixel region was selected. A fluorescence filter set (Semrock, Rochester, NY, LF405/488/561/635-A-000) was used to select and separate the excitation and emission lights in the microscope. Additional emission filters (Semrock, BLP01-647R-25) were used in the detection path to further clean the fluorescent light spectrally for the reduction of spectral crosstalk.

During the measurements, the perfect focus system of the microscope was used to keep the sample in focus with a precision of <30 nm. The storage buffer on the sample was replaced with a special switching buffer (20). Typically, 10,000 frames were captured with an exposure time of 30 ms. Reference images with full size FOV were captured at low intensity when the majority of fluorescent molecules were still active and the overall structure of the labeled sample could be visualized.

The captured and stored image stacks were evaluated and analyzed by rainSTORM localization software (21). The individual images of single molecules were fitted with a Gaussian point spread function and their center positions were associated with the position of the fluorescent molecule. Localizations were filtered via their intensity, ellipticity and standard deviation values. Localizations with precisions of <45 nm were only used to form the final image. The estimated mean precision of the accepted localizations was 19 nm. Mechanical drift introduced by either the mechanical movement of the sample or thermal effects was analyzed and reduced by means of a blind drift correction algorithm. Spatial coordinates of the localized molecules were stored and the final super-resolved image was visualized. The multicolor merged images were generated by ImageJ software.

### *In vivo* Experiments

#### Mouse Model

B16F1 melanoma cells were administrated intravenously (1 × 10^5^ cell/100 μl) to 6–8 week old female C57BL/6N mice (Charles River Laboratories). One week later, tumor bearing mice were randomized and divided into 3 groups (*n* = 10/group). Mice were injected intravenously with control buffer (100 μl), exosome exposed MSCs (1 × 10^5^ cell/100 μl) or exosomes (40 μg/100 μl) 7, 8, 9, 10, 11 days after the tumor injection (Table S2). One week after the first MSC administration, 3 animals/group were euthanized, their lungs were removed, photographed and stored at −80°C for further protein, mRNA and histological analyses. The remained mice were observed for 10 more days. At the end point, the animals were euthanized, and the tumor metastases were investigated not only in their lung, but also in their entire body and removed for histological analysis. Experiments were repeated 3 times. All animal experiments were performed in accordance with national (1998. XXVIII; 40/2013) and European (2010/63/EU) animal ethics guidelines. The experimental protocols were approved by the Animal Experimentation and Ethics Committee of the Biological Research Centre of the Hungarian Academy of Sciences and the Hungarian National Animal Experimentation and Ethics Board (clearance number: XVI./03521/2011 and XV./78/2018).

#### Tumor Coverage

Tumor coverage of lungs was determined by the analysis of acquired images using the ImageJ software. The area of tumors and the healthy regions was measured and the mean percentage, SD, and *p* values were calculated in Microsoft Excel.

#### Quantification of Metastases Associated Vessel Diameters

From native animal lungs 4 μm criostat sections were made on silanized slides, than fixed and retrieved by Fix and Perm A-B solution (Thermo Fisher Scientific, USA) for 20 min. Sections were counterstained by conventional hematoxilin for 30 min than washed in tap water and coverslipped. Sections were digitalized by automatic slide-scanner (3DHistech, Hungary), using the software 3DHISTECH Pannoramic Viewer (3DHistech, Hungary). Strict tumor border was carefully marked then vessel diameters were measured.

#### Cytokine and Chemokine Array

Lung samples were lysed in NP40 cell lysis buffer (Thermo Fisher Scientific, USA) and protein content was measured by the Pierce BCA Protein Assay kit (Thermo Fisher Scientific, USA). Expression levels of different cytokines in pooled lung specimens were determined using Mouse Cytokine Array Panel A (R&D Systems, Cat. no. ARY006), according to the manufacturer's instructions. Immunoreactive signals were detected by using LI-COR ODYSSEY Fc (Dual-mode imaging system) imager followed by analysis with Image Studio Lite v5.2.

#### Custom TaqMan Array Panels

QRT-PCR was performed on an ABI Prism 7000 sequence detection system (Applied Biosystems) using specific pre-designed customized 96-well TaqMan Array oncogene panels (Thermo Fisher Scientific) containing 44 specifically selected primers and probes according to extensive literature work. Total RNA was isolated using TRIzol (Thermo Fisher Scientific), DNase treatment was performed according to the manufacturer's protocol, and then total RNA was transcribed into cDNA using High Capacity cDNA Kit (Thermo Fisher Scientific). PCR amplification was performed using TaqMan primers and probes and thermal cycle conditions were set as follows: 2 min at 50°C, 10 min at 95°C, and 40 cycles of 15 s at 95°C and 1 min at 60°C. As internal control transcripts of ACTB (Assay ID: Mm00607939_s1), GAPDH (Assay ID: Mm99999915_g1), and PPIA (Assay ID: Mm02342430_g1) were determined. The amount of the transcripts was normalized to those of the housekeeping genes using the ΔCt method. Finally the results were normalized to the expression of the vehicle control (ΔΔCt method).

For relationship discovery Hierarchical Cluster Analysis was performed by R software. In detail, the “bottom up” agglomerative hierarchical clustering strategy was used and results represented in a tree-based dendrogram [For refer the R software: (22)].

#### Network Representation by Ingenuity Pathway Analysis

According to literature data, we established a protein network from the *in vivo* overexpressed genes. A custom graphical representation of this network was generated using the Path Explorer tool of the IPA Path Designer. Genes are represented as red nodes, using various shapes that represent the functional class of the gene product. In a few cases proteins are substituted with the complex, which they are involved in. To identify the potential exosomal factors, which can induce the activation of the network, a list was generated from the exosomal proteins detected by LC-MS/MS and the exosomal miRNAs identified by SOLiD sequencing. The Grow tool in the IPA Path Designer revealed significant interactions between the network and the generated list (the interacting exosomal factors are listed in gray boxes and the types of relationships are indicated in parentheses). During construction in the IPA, the significance level was set to “experimentally observed” data to avoid the representation of predicted, unproven interactions.

### Statistical Analysis

All of the data are presented as the mean ± SD or SEM and represent minimum of three independent experiments. Statistical parameters including statistical analysis, statistical significance, and n value are reported in the figure legends. For *in vivo* experiments *n* = number of animals. For statistical comparison, we performed two-tailed Student's *t*-test. A value of *p* < 0.05 was considered significant [represented as ^*^p < 0.05, not significant (n.s.)].

## Results

### Isolated Vesicles Show Exosomal Properties

**Figure 1:**
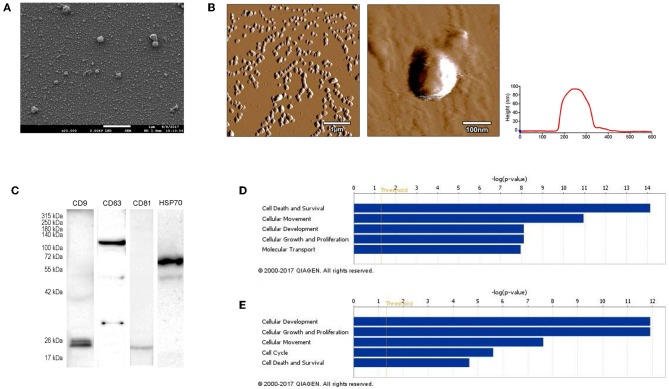
Characterization of B16F1 melanoma cell culture-derived exosomes. **(A)** Scanning electron micrograph of melanoma exosomes. **(B)** Atomic force microscopy images of exosomes. Left and middle pictures represent the shape and surface topography of vesicles, right graph represents height profile. **(C)** Western blot analysis of common exosome markers (CD9, CD63, CD81, and HSP70). **(D,E)** Top 5 molecular and cellular functions identified by Ingenuity Pathway Analysis of exosomal proteins and miRNAs.

First, we isolated extracellular vesicles from B16F1 mouse melanoma cells. As it was shown by SEM and AFM (Figures 1A,B), the isolated fraction indeed contained exosomes as the particles were cap-shaped, and their size was within the 40–120 nm range. Then, presence of molecules (CD9, CD63, CD81, and HSP70), characteristic for exosomes (4, 17) was assessed by Western blotting (Figure 1C).

Exosomes were then subjected to large-scale analysis to determine their protein and miRNA profiles. Whole proteome analysis (using LC-MS/MS) and bioinformatics tools (used to identify elements of the exosome's proteomics spectrum in the UniProt database, and to compare the findings with entries of the ExoCarta database) revealed that 95 distinct proteins were identified in melanoma exosomes (Table 1). These proteins exhibited 86.3% overlap with molecules listed as characteristic exosome protein markers in ExoCarta. Further, miRNA sequencing (using SOLiD 5500xl technology) identified 168 known miRNA elements (Table 2) which, similarly to the proteomics data, exhibited a large (93.5%) overlap with molecules listed as characteristic exosome miRNA markers in ExoCarta.

**Table 1:** List of exosomal proteins identified by LC-MS/MS.

**ID**	**Symbol**	**Entrez gene name**
P97857	ADAMTS1	ADAM metallopeptidase with thrombospondin type 1 motif 1
Q3TNX8	ADAMTS4	ADAM metallopeptidase with thrombospondin type 1 motif 4
Q640N1	AEBP1	AE binding protein 1
P05064	ALDOA	Aldolase, fructose-bisphosphate A
P97429	ANXA4	Annexin A4
P48036	ANXA5	Annexin A5
P08226	APOE	Apolipoprotein E
Q3TWT5	ASAH1	N-acylsphingosine amidohydrolase 1
Q3TXF9	ATP1A1	ATPase Na^+^/K^+^ transporting subunit alpha 1
P97370	ATP1B3	ATPase Na^+^/K^+^ transporting subunit beta 3
Q1XID4	ATP6AP2	ATPase H^+^ transporting accessory protein 2
Q9JL18	BACE2	Beta-site APP-cleaving enzyme 2
O55107	BSG	Basigin (Ok blood group)
Q8R2Q8	Bst2	Bone marrow stromal cell antigen 2
Q9WVT6	CA14	Carbonic anhydrase 14
P41731	CD63	CD63 molecule
P35762	CD81	CD81 molecule
P10605	CTSB	Cathepsin B
P18242	CTSD	Cathepsin D
P29812	DCT	Dopachrome tautomerase
P57776	EEF1D	Eukaryotic translation elongation factor 1 delta
Q3UAM9	ENG	Endoglin
P17182	ENO1	Enolase 1
P19096	FASN	Fatty acid synthase
P30416	FKBP4	FK506 binding protein 4
P11276	FN1	Fibronectin 1
P09528	FTH1	Ferritin heavy chain 1
P16858	GAPDH	Glyceraldehyde-3-phosphate dehydrogenase
P08752	GNAI2	G protein subunit alpha i2
Q3TAV1	GPNMB	Glycoprotein nmb
P19157	GSTP1	Glutathione S-transferase pi 1
P11499	HSP90AB1	Heat shock protein 90 alpha family class B member 1
B1B0C7	HSPG2	Heparan sulfate proteoglycan 2
Q9CQW9	IFITM3	Interferon induced transmembrane protein 3
G3UYZ1	IGSF8	Immunoglobulin superfamily member 8
Q91VK4	ITM2C	Integral membrane protein 2C
P02468	LAMC1	Laminin subunit gamma 1
Q60961	LAPTM4A	Lysosomal protein transmembrane 4 alpha
P35951	LDLR	Low density lipoprotein receptor
Q07797	LGALS3BP	Galectin 3 binding protein
Q3U2W5	LGALS8	Galectin 8
P16056	MET	MET proto-oncogene, receptor tyrosine kinase
P21956	MFGE8	Milk fat globule-EGF factor 8 protein
Q2TA50	MLANA	Melan-A
Q6NVG5	MREG	Melanoregulin
Q9EPX2	PAPLN	Papilin, proteoglycan like sulfated glycoprotein
Q3UIP2	PCOLCE	Procollagen C-endopeptidase enhancer
Q811J2	LOC72520	LOC72520 protein
Q80Y09	PDCD6IP	Programmed cell death 6 interacting protein
P62962	PFN1	Profilin 1
P09411	PGK1	Phosphoglycerate kinase 1
P52480	PKM	Pyruvate kinase, muscle
Q9CZB2	PMEL	Premelanosome protein
P17742	PPIA	Peptidylprolyl isomerase A
P35700	PRDX1	Peroxiredoxin 1
Q61171	PRDX2	Peroxiredoxin 2
Q543S0	PRELP	Proline and arginine rich end leucine rich repeat protein
P53994	RAB2A	RAB2A, member RAS oncogene family
Q8CCG5	RALB	RAS like proto-oncogene B
O89086	RBM3	RNA binding motif (RNP1, RRM) protein 3
P35980	RPL18	Ribosomal protein L18
Q3U5P4	SCPEP1	Serine carboxypeptidase 1
O08992	SDCBP	Syndecan binding protein
Q0VGP2	SEMA3B	Semaphorin 3B
P32261	SERPINC1	Serpin family C member 1
P10852	SLC3A2	Solute carrier family 3 member 2
Q3UQM7	SLC7A5	Solute carrier family 7 member 5
O09044	SNAP23	Synaptosome associated protein 23
Q64337	SQSTM1	Sequestosome 1
Q8CI59	STEAP3	STEAP3 metalloreductase
Q3TDG9	STX12	Syntaxin 12
O70439	STX7	Syntaxin 7
P40749	SYT4	Synaptotagmin 4
O88968	TCN2	Transcobalamin 2
Q542D9	TFRC	Transferrin receptor
P39876	TIMP3	TIMP metallopeptidase inhibitor 3
Q4FJX7	TINAGL1	Tubulointerstitial nephritis antigen like 1
Q9DCS1	TMEM176A	Transmembrane protein 176A
Q9R1Q6	TMEM176B	Transmembrane protein 176B
Q9CZX7	TMEM55A	Transmembrane protein 55A
Q9QY73	TMEM59	Transmembrane protein 59
O88746	TOM1	Target of myb1 membrane trafficking protein
O89023	TPP1	tripeptidyl peptidase 1
Q3UCW0	TSG101	Tumor susceptibility 101
Q4FJW7	TSPAN4	Tetraspanin 4
Q8BJU2	TSPAN9	Tetraspanin 9
P11344	TYR	Tyrosinase
P07147	TYRP1	Tyrosinase related protein 1
O70404	VAMP8	Vesicle associated membrane protein 8
Q8R0J7	VPS37B	VPS37B, ESCRT-I subunit
Q8R105	VPS37C	VPS37C, ESCRT-I subunit
O88384	VTI1B	Vesicle transport through interaction with t-SNAREs 1B
A8DUQ1	HBBT1	Beta-globin
P70356	MELA	Gag-pol poliprotein
P70355	MELA	Envelope protein

**Table 2:** List of exosomal miRNAs identified by SOLiD 5500xl technology.

**Symbol**	**Seed regio**	**ID**
let-7a-3p	UAUACAA	mmu-let-7a-1-3p
		mmu-let-7b-3p
		mmu-let-7c-2-3p
		mmu-let-7f-1-3p
let-7a-5p	GAGGUAG	mmu-let-7a-5p
		mmu-let-7b-5p
		mmu-let-7c-5p
		mmu-let-7d-5p
		mmu-let-7e-5p
		mmu-let-7f-5p
		mmu-let-7g-5p
		mmu-mir-98-5p
let-7d-3p	UAUACGA	mmu-let-7d-3p
let-7i-3p	UGCGCAA	mmu-let-7i-3p
miR-100-5p	ACCCGUA	mmu-mir-99a-5p
		mmu-mir-99b-5p
miR-101-3p	ACAGUAC	mmu-mir-101a-3p
miR-103-1-5p	GCUUCUU	mmu-mir-107-5p
miR-103-3p	GCAGCAU	mmu-mir-103-3p
		mmu-mir-107-3p
miR-10a-5p	ACCCUGU	mmu-mir-10a-5p
		mmu-mir-10b-5p
miR-1191a	AGUCUUA	mmu-mir-1191a
miR-1249-3p	CGCCCUU	mmu-mir-1249-3p
miR-125b-5p	CCCUGAG	mmu-mir-125a-5p
		mmu-mir-125b-5p
		mmu-mir-351-5p
miR-126a-5p	AUUAUUA	mmu-mir-126a-5p
miR-128-3p	CACAGUG	mmu-mir-128-3p
miR-129-1-3p	AGCCCUU	mmu-mir-129-1-3p
		mmu-mir-129-2-3p
miR-129b-5p	CUUUUUG	mmu-mir-129b-5p
miR-130a-3p	AGUGCAA	mmu-mir-130a-3p
		mmu-mir-130b-3p
		mmu-mir-301a-3p
		mmu-mir-301b-3p
miR-130a-5p	CUCUUUU	mmu-mir-130a-5p
miR-130b-5p	CUCUUUC	mmu-mir-130b-5p
miR-132-3p	AACAGUC	mmu-mir-132-3p
miR-132-5p	ACCGUGG	mmu-mir-132-5p
miR-135a-5p	AUGGCUU	mmu-mir-135a-5p
miR-138-5p	GCUGGUG	mmu-mir-138-5p
miR-139-5p	CUACAGU	mmu-mir-139-5p
miR-140-3p	ACCACAG	mmu-mir-140-3p
miR-140-5p	AGUGGUU	mmu-mir-140-5p
miR-142-3p	GUAGUGU	mmu-mir-142a-3p
miR-143-5p		mmu-mir-143-5p
miR-144-3p	ACAGUAU	mmu-mir-144-3p
miR-144-5p	GAUAUCA	mmu-mir-144-5p
miR-145-5p	UCCAGUU	mmu-mir-145a-5p
miR-146a-5p	GAGAACU	mmu-mir-146a-5p
miR-148a-3p	CAGUGCA	mmu-mir-148b-3p
miR-151-3p	UAGACUG	mmu-mir-151-3p
miR-15a-3p	AGGCCAU	mmu-mir-15a-3p
miR-15b-3p	GAAUCAU	mmu-mir-15b-3p
miR-16-2-3p	CCAAUAU	mmu-mir-16-2-3p
miR-16-5p	AGCAGCA	mmu-mir-15a-5p
		mmu-mir-15b-5p
		mmu-mir-16-5p
		mmu-mir-195a-5p
		mmu-mir-322-5p
		mmu-mir-497a-5p
miR-17-3p	CUGCAGU	mmu-mir-17-3p
miR-17-5p	AAAGUGC	mmu-mir-106b-5p
		mmu-mir-17-5p
		mmu-mir-20a-5p
		mmu-mir-93-5p
miR-181a-1-3p	CCAUCGA	mmu-mir-181a-1-3p
miR-181a-5p	ACAUUCA	mmu-mir-181a-5p
		mmu-mir-181b-5p
		mmu-mir-181c-5p
		mmu-mir-181d-5p
miR-1827	GAGGCAG	mmu-mir-709
miR-1839-3p	GACCUAC	mmu-mir-1839-3p
miR-185-5p	GGAGAGA	mmu-mir-185-5p
miR-186-5p	AAAGAAU	mmu-mir-186-5p
miR-187-3p	CGUGUCU	mmu-mir-187-3p
miR-188-3p	UCCCACA	mmu-mir-188-3p
miR-188-5p	AUCCCUU	mmu-mir-188-5p
miR-18a-5p	AAGGUGC	mmu-mir-18a-5p
miR-191-5p	AACGGAA	mmu-mir-191-5p
miR-193a-3p	ACUGGCC	mmu-mir-193a-3p
miR-1981-3p	AUCUAAC	mmu-mir-1981-3p
miR-199a-3p	CAGUAGU	mmu-mir-199a-3p
		mmu-mir-199b-3p
miR-199a-5p	CCAGUGU	mmu-mir-199a-5p
		mmu-mir-199b-5p
miR-19b-3p	GUGCAAA	mmu-mir-19a-3p
		mmu-mir-19b-3p
miR-204-5p	UCCCUUU	mmu-mir-211-5p
miR-21-5p	AGCUUAU	mmu-mir-21a-5p
miR-210-3p	UGUGCGU	mmu-mir-210-3p
miR-210-5p	GCCACUG	mmu-mir-210-5p
miR-219a-5p	GAUUGUC	mmu-mir-219a-5p
miR-22-3p	AGCUGCC	mmu-mir-22-3p
miR-22-5p	GUUCUUC	mmu-mir-22-5p
miR-221-3p	GCUACAU	mmu-mir-222-3p
miR-223-3p	GUCAGUU	mmu-mir-223-3p
miR-224-5p	AAGUCAC	mmu-mir-224-5p
miR-23a-3p	UCACAUU	mmu-mir-23a-3p
		mmu-mir-23b-3p
miR-24-1-5p	UGCCUAC	mmu-mir-24-2-5p
miR-24-3p	GGCUCAG	mmu-mir-24-3p
miR-26a-5p	UCAAGUA	mmu-mir-26a-5p
miR-26a-5p	UCAAGUA	mmu-mir-26a-5p
		mmu-mir-26b-5p
miR-27a-3p	UCACAGU	mmu-mir-27a-3p
		mmu-mir-27b-3p
miR-29a-5p	CUGAUUU	mmu-mir-29a-5p
miR-29b-1-5p	CUGGUUU	mmu-mir-29b-1-5p
miR-29b-3p	AGCACCA	mmu-mir-29a-3p
		mmu-mir-29b-3p
		mmu-mir-29c-3p
miR-3065-5p	CAACAAA	mmu-mir-3065-5p
miR-30c-5p	GUAAACA	mmu-mir-30a-5p
		mmu-mir-30b-5p
		mmu-mir-30c-5p
		mmu-mir-30d-5p
		mmu-mir-30e-5p
miR-31-3p	GCUAUGC	mmu-mir-31-3p
miR-31-5p	GGCAAGA	mmu-mir-31-5p
miR-3176	CUGGCCU	mmu-mir-378d
miR-324-5p	GCAUCCC	mmu-mir-324-5p
miR-328-3p	UGGCCCU	mmu-mir-328-3p
miR-329-3p	ACACACC	mmu-mir-362-3p
miR-33-5p	UGCAUUG	mmu-mir-33-5p
miR-330-5p	CUCUGGG	mmu-mir-326-3p
miR-331-3p	CCCCUGG	mmu-mir-331-3p
miR-339-5p	CCCUGUC	mmu-mir-339-5p
miR-340-3p	CCGUCUC	mmu-mir-340-3p
miR-344a-5p	CAGGCUC	mmu-mir-484
miR-345-5p	CUGACCC	mmu-mir-345-5p
miR-3473b	GGCUGGA	mmu-mir-3473b
		mmu-mir-3473e
miR-34a-5p	GGCAGUG	mmu-mir-34a-5p
		mmu-mir-34b-5p
		mmu-mir-34c-5p
miR-34c-3p	AUCACUA	mmu-mir-34b-3p
miR-350	UCACAAA	mmu-mir-350-3p
miR-361-5p	UAUCAGA	mmu-mir-361-5p
miR-362-5p	AUCCUUG	mmu-mir-362-5p
miR-374b-5p	UAUAAUA	mmu-mir-374b-5p
miR-378a-3p	CUGGACU	mmu-mir-378a-3p
		mmu-mir-378c
miR-378a-5p	UCCUGAC	mmu-mir-378a-5p
miR-3909	GUCCUCU	mmu-mir-877-3p
miR-423-3p	GCUCGGU	mmu-mir-423-3p
miR-423-5p	GAGGGGC	mmu-mir-423-5p
miR-425-5p	AUGACAC	mmu-mir-425-5p
miR-451a	AACCGUU	mmu-mir-451a
miR-501-5p	AUCCUUU	mmu-mir-501-5p
miR-503-5p	AGCAGCG	mmu-mir-503-5p
miR-532-5p	AUGCCUU	mmu-mir-532-5p
miR-542-3p	GUGACAG	mmu-mir-542-3p
miR-574-5p	GAGUGUG	mmu-mir-574-5p
miR-582-5p	UACAGUU	mmu-mir-582-5p
miR-652-3p	AUGGCGC	mmu-mir-652-3p
miR-670-5p	UCCCUGA	mmu-mir-670-5p
miR-700-5p	AAGGCUC	mmu-mir-700-5p
miR-744-3p	UGUUGCC	mmu-mir-744-3p
miR-744-5p		mmu-mir-744-5p
miR-7a-5p	GGAAGAC	mmu-mir-7a-5p
miR-872-3p	GAACUAU	mmu-mir-872-3p
miR-872-5p	AGGUUAC	mmu-mir-872-5p
miR-9-5p	CUUUGGU	mmu-mir-9-5p
miR-92a-3p	AUUGCAC	mmu-mir-25-3p
		mmu-mir-32-5p
		mmu-mir-92a-3p

To uncover the functional significance of the proteomics and miRNA sequencing data, IPA was employed. This analysis has shown that the identified proteins most probably participated in cellular and molecular processes such as “Cell Death and Survival,” “Cellular Movement,” “Cell-to-Cell Signaling and Interaction,” “Cellular Growth and Proliferation,” and “Cell Morphology” (*P*_range_ = 7.53 × 10^−15^-9.32 × 10^−4^ significance range) (Figure 1D). Very similarly to these data, functions of the identified miRNAs were suggested to be linked to mechanisms of “Cellular Development,” “Cellular Growth and Proliferation,” “Cellular Movement,” “Cell Cycle,” and “Cell Death and Proliferation” (*P*_range_ = 1.25 × 10^−12^-4.88 × 10^−2^ significance range) (Figure 1E).

### Tumor Exosome Exposure Resulted in Oncogenic Reprogramming of MSCs *in vitro*

Then, we investigated the effect of exosomes on biological processes (e.g., proliferation, survival, malignant transformation, etc.) of MSCs, which are generally considered as proper *in vitro* models of tumor stroma (12). For these experiments, MSC cultures were initiated from mouse abdominal adipose tissue (17) and were subjected to melanoma exosome treatment.

**Figure 2:**
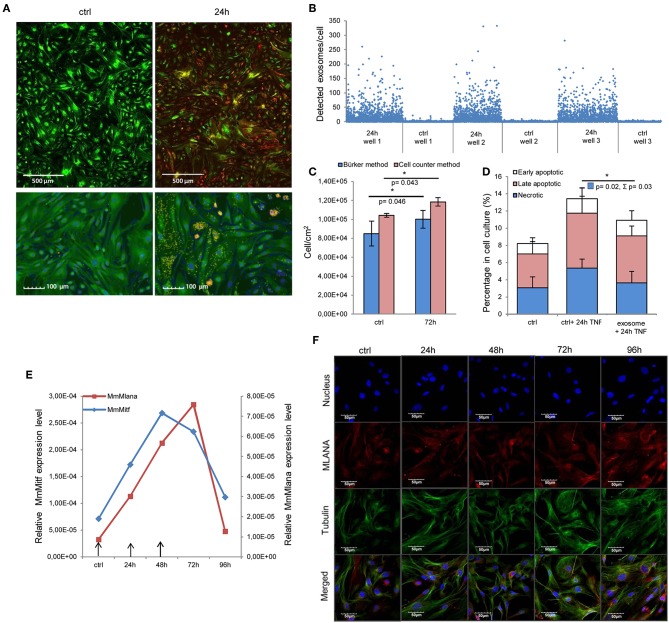
Internalized melanoma exosomes induce malignant transformation of the recipient MSCs. **(A)** Fluorescent images of exosome uptake by MSCs. DiO (green lipid dye)-labeled cells were exposed to Dil (red lipid dye)-labeled exosomes for 24 h and fixed in 4% PFA. Control cells were treated in the same manner without exosomes. Pictures were acquired by an Operetta high content screening system (Perkin Elmer). Lower two images represent an experiment, where nuclei were counterstained with DAPI. Exosomes were detected with a customized version of A-trous wavelet transform, and were highlighted with yellow. **(B)** Quantitative analysis of exosome uptake. The graph shows the number of detected exosomes (Y axis) in each cell (X axis) in three exosome-exposed (24 h) and three control cell cultures (ctrl). **(C)** Cell proliferation assay of exosome-exposed MSCs. Cells were plated at 1 × 10^4^ cell/cm^2^ density in control and exosome-exposed cultures, and 72 h after the exosome treatment, the cell number was determined by manual counting and by an automated cell counter. Both methods showed a significantly increased cell proliferation of exosome-treated cells. Results are presented as mean ± SD (*n* = 3). **(D)** Apoptosis analysis of exosome-exposed MSCs by flow cytometry. Exosome-pre-exposed cells were treated by 100 ng/ml TNFα for 24 h, stained with Annexin V-FITC and propidium iodide. They were analyzed by flow cytometry both in the case of the TNFα-treated and the untreated control cell cultures. The graph represents the percentage of early apoptotic, late apoptotic and necrotic cells. Both TNFα-induced total cell death (*Σ*) and necrosis were significantly lower (*Σ*p = 0.03 and *p* = 0.02, respectively) in exosome-pretreated cell cultures compared to the corresponding control cells. Results are presented as mean + SD (*n* = 3). **(E)** QRT-PCR analysis of Mlana and Mitf in MSC cultures treated by exosomes in every 24 h indicated by arrows in the graph. The expression of both mRNAs increased after exosome exposure, but they showed different kinetics. **(F)** Fluorescent immunocytochemistry of MLANA in exosome-exposed MSC cultures using a primary rabbit antibody to MLANA and a secondary AlexaFluor555-conjugated antibody to rabbit IgG (red). α-tubulin network of cells was directly labeled by an AlexaFluor488-conjugated antibody (green) and the nuclei were stained with DAPI (blue).

First, we assessed whether exosomes were internalized by MSCs. High-throughput microscopy showed that MSCs [labeled green by the DiOC_18_(3) lipid dye] indeed took up exosomes [labeled red by the DilC_18_(3) lipid dye] as early as 1–2 h after application (Figure S1). Importantly, after 24 h, the majority of MSCs were loaded by exosomes (Figures 2A,B). Indeed, image analysis and statistical evaluation revealed a 91% internalization efficacy. This suggests that functional alterations demonstrated by this study were due to exosome-induced cell-population, and not individual cell level effects.

Then, we determined whether the internalized exosomes could induce a melanoma-like malignant transformation of the MSCs. By employing two complementary cell-counting methods, we found that proliferation rate of the MSCs significantly accelerated 72 h after exosome exposure (Figure 2C). By flow cytometry, we also showed that the exosome-treated MSCs exhibited a partial resistance to the cell death-inducing effects of 100 ng/ml tumor necrosis factor-α (TNFα) as the fraction of the dead cells was significantly decreased in these cultures (Figure 2D).

Since the exosomes were isolated from melanoma cells, we were then intrigued to uncover whether the above alterations (which all argue for the malignant transformation of the susceptible cells) also resulted in *de novo* appearance of melanoma-specific features in the transformed MSCs. To answer this question, expression of the melanoma-specific markers MLANA and MITF were investigated. By QRT-PCR, we found that mRNA transcript levels of both markers elevated markedly in MSCs upon exosome treatment (Figure 2E and Figure S2), albeit the kinetics of elevation of the two molecules were slightly different. Moreover, in good accordance with the mRNA data, immunofluorescence labeling showed that exosome exposure markedly increased the expression of MLANA at the protein level as well (Figure 2F).

Next, we assessed whether the above effects of exosomes inducing a malignant-like transformation of the MSCs was accompanied by a cellular-molecular oncogenic reprogramming of the target cells. Naïve MSCs were exposed to a standardized volume of exosomes for various time intervals (to avoid the experimental fluctuations, we collected pooled samples from multiple independent *in vitro* experiments). Then, samples were subjected to QRT-PCR analysis using a self-designed panel of 40 oncogenes and tumor suppressor genes which were previously suggested to play a role in melanoma progression (Table 3).

**Table 3:** List of genes investigated by a self-designed oncopanel.

**Gene**	**Protein**	**Function**	**References**
Alcam	ALCAM (CD166) Activated leukocyte cell adhesion molecule	Plays an important role in human malignant melanoma progression and formation of locoregional and distant metastases	(23)
Bmi1	BMI1 B cell-specific Moloney murine leukemia virus integration site 1	Induces an invasive signature in melanoma that promotes metastasis and chemoresistance	(24)
Cd44	CD44	Is a CD44s interaction with HA plays a crucial role in cell invasiveness	(25)
Eng	ENG Endoglin (CD105)	Has a crucial role in angiogenesis, important protein for tumor growth, survival, and metastasis of cancer cells to other locations in the body	(26)
Flot2	FLOT1 Flotillin-2	Is associated with melanoma progression	(27)
Itga2	ITGA2 Integrin alpha2	Is associated with increased risk of melanoma	(28)
Itga4	ITGA4 Integrin alpha 4	α4β1 integrin plays an important role in metastasis of malignant melanoma	(28)
Itga6	ITGA6 Integrin alpha 6	α6β1 integrin as a laminin receptor expression is associated with invasive potential in a highly metastatic melanoma cell line	(28)
Itgb1	ITGB1 Integrin beta-1 (CD29)	α4β1 integrin plays an important role in metastasis of malignant melanoma	(28)
Kit	KIT (CD117) Mast/stem cell growth factor receptor (SCFR)	c-Kit signaling activates the MAPK and PI3K signaling cascades	(29)
Muc1	MUC1 Mucin1 cell surface associated	Promotes melanoma migration through the Akt signaling pathway	(29)
Pecam1	PECAM1 Platelet endothelial cell adhesion molecule (CD31)	Can play multiple roles in diverse processes related to melanoma development, dormancy, migration/invasion, and angiogenesis	(30)
Prom1	Prominin-1 (CD133)	Is a melanoma stem cell marker	(31)
Thy	CD90	Is a cell adhesion molecule. Melanoma cells use Thy-1 on endothelial cells for metastasis formation	(32)
Cdc42	CDC42 Cell division control protein 42 homolog	Is vital for the transforming Ras signal emanating from endomembranes	(33)
Tiam1	TIAM1 T-cell lymphoma invasion and metastasis 1	Has crucial roles in regulation of the actin cytoskeleton, cell migration, cell cycle progression, gene transcription, and cell adhesion	(34)
Bcl2	BCL-2 B-cell lymphoma 2	Plays a pivotal role in the regulation of molecules associated with the migratory and invasive phenotype, contributing, in cooperation to hypoxia, to tumor progression	(29)
Bax	BAX Bcl-2-associated X protein	Plays a crucial role in apoptotic cell death induced, the Bax/Bcl-2 ratio determines the susceptibility of melanoma cells	(35)
Casp9	CASP9 Caspase-9	Is linked to the mitochondrial death pathway	(35)
Casp8	CASP8 Caspase-8	Plays a central role in the execution-phase of cell apoptosis	(36)
Cdk4	CDK4 Cyclin-dependent kinase 4	Promotes cell-cycle progression and inhibit both cell senescence and apoptosis	(29)
Elk1	ELK1 ETS domain-containing protein Elk-1	Is a member of ETS oncogene family, transcription activator	(37)
Ets1	ETS1 E26 transformation-specific	Is required for migration in cell lines with an active RAS*/*ERK signaling pathway	(29)
Hgf	HGF Hepatocyte growth factor	Can activate the MAP-kinase pathway, which is upregulated in the majority of melanoma, through the proto-oncogene c-MET	(38)
Jak2	JAK2 Janus kinase 2	Is an activator of transcription (STAT) pathway is thought to play a central role in melanoma cell biology	(39)
Met	MET Hepatocyte growth factor receptor	Induces several biological responses that collectively give rise to a program known as invasive growth	(40)
Myb	MYB transcriptional activator Myb	Is a transcription factor. Among other genes, MYB regulates the transcription of the Kit, Bcl2, Ets-2, and N-Ras	(41)
Nras	NRAS Neuroblastoma RAS viral oncogene homolog	Recruits and stimulates a number of intracellular signaling pathways including the Raf/MEK/ERK mitogen activated protein kinase (MAPK) pathway, the PI3K/AKT pathway	(29)
Stat3	STAT3 Signal transducer and activator of transcription 3	Promotes transcription of many genes that involve in melanoma metastasis	(42)
Kitl	KIT-ligand Stem cell factor (CD117)	Is a cytokine that binds to the c-KIT receptor. This cytokine plays an important role in melanogenesis	(29)
Rb1	RB1 Retinoblastoma 1 protein	Is a tumor suppressor protein that is dysfunctional in several major cancers	(43)
Pik3ca	PI3K Phosphatidylinositide 3-kinases	Is a PI3K/AKT pathway play a pivotal role in tumor development, growth, and metastasis of melanoma	(29)
Raf1	RAF1 Proto-oncogene serine/threonine-protein kinase	Is a crucial regulators of the ERK MAP kinase signaling cascade	(44)
Mtor	mTOR Serine/threonine-protein kinase	Mechanistic target of rapamycin, is a serine/threonine protein kinase that regulates cell growth, cell proliferation, cell motility, cell survival	(29)
Akt1	AKT Protein kinase B	Plays a key role in multiple cellular processes such as glucose metabolism, apoptosis, cell proliferation, transcription, and cell migration	(29)
Map2k1	MEK1 Dual specificity mitogen-activated protein kinase kinase 1	Is an essential component of the MAP kinase signal transduction pathway, this kinase is involved in many cellular processes such as proliferation, differentiation, transcription regulation, and development	(29)
Map2k2	MEK2 Dual specificity mitogen-activated protein kinase kinase 2	Plays a critical role in mitogen growth factor signal transduction. It phosphorylates and thus activates MAPK1/ERK2 and MAPK2/ERK3	(29)
Mapk3	ERK1 Extracellular-signal-regulated kinases	Ras-Erk1/2 is a key regulator pathway in melanoma cell proliferation	(29)
Mapk1	ERK2 Mitogen-activated protein kinase 1	Ras-Erk1/2 is a key regulator pathway in melanoma cell proliferation	(29)
Rac1	RAC1 Ras-related C3 botulinum toxin substrate 1	Functions in multiple signaling pathways are leading to cell adhesion, migration, proliferation, and transformation	(45)

**Figure 3:**
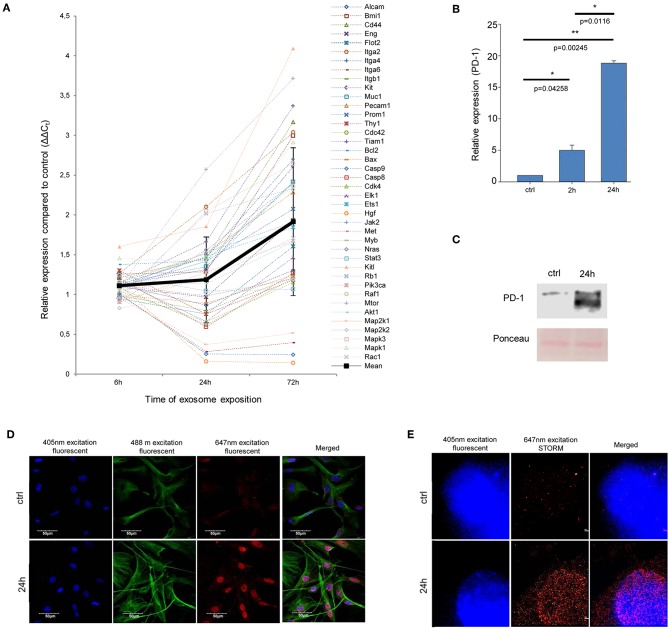
Exosome re-educated MSCs show oncogene dominance and PD-1 expression. **(A)** QRT-PCR analysis of 40 tumor-related genes in exosome-exposed MSCs using a self-designed panel. The graph shows the relative expression values for each gene after 6, 24, and 72 h of exosome exposure. The trend line of altered gene expression pattern (indicated by thick black line) shows an increasing tendency over time (mean ± SD). **(B)** QRT-PCR analysis of PD-1 in MSCs after 2 and 24 h of exosome exposure. The graph represents mean + SEM (*n* = 3). **(C)** Representative immunoblot of PD-1 protein expression in control and exosome-exposed MSCs after 24 h of exosome treatment. **(D,E)** Fluorescent immunocytochemistry of PD-1 in 24 h exosome-exposed MSC cultures, using a primary rat antibody to PD-1 and a secondary AlexaFluor647-conjugated antibody to rat IgG (red). Nuclei were stained with DAPI. **(D)** α-tubulin network of cells was directly labeled by an AlexaFluor488-conjugated antibody (green). Images were acquired by confocal microscopy. **(E)** Images were taken by STORM super-resolution microscopy. STORM super-resolution imaging of PD-1 revealed that PD-1 was localized mostly around the nucleus, which were blurred and were not resolvable using diffraction-limited confocal microscopy **(D)** of the same region.

As shown in Figure 3A, gene expression pattern of MSCs exposed to melanoma exosomes, exhibited a clear oncogenic dominance (compared to the non-exposed cells). This was verified by statistical analysis of the mean relative gene expression levels of all molecules investigated. Statistically higher values were obtained in the case of exosome-treated cells (*p* = 1.9 × 10^−5^, *p* = 0.031 and *p* = 2.3 × 10^−8^ for the 6, 24, and 72 h time points, respectively).

As mentioned above, Kleffel et al. has recently shown that melanoma cell subpopulations which overexpress PD-1, quite intriguingly exhibit remarkably increased invasiveness and aggressive growth properties (15). However, the authors did not define the factor(s) which induced the above PD-1 overexpression. Since the above finding strongly suggested an “MSC re-education” capacity of melanoma exosomes to induce malignant-like behavior, we next assessed the expression of PD-1 in MSC cultures.

As expected, only insignificant PD-1 expression (both at the mRNA and protein levels) could be identified in control, non-treated MSCs. In contrast, a marked, significant, and time-dependent elevation of PD-1 expression was detected upon exosome treatment by QRT-PCR (Figure 3B), Western blot and an immunocytochemical analysis (Figures 3C,D). Further, by employing super-resolution microscopy, we were able to identify a dramatic upregulation of PD-1 at the single molecular level in exosome-treated MSCs (Figure 3E).

Importantly, since proteomics analysis did not identify the presence of PD-1 in exosomes, these data suggest that the high PD-1 protein content in exosome-exposed MSCs was a result of *de novo* induction and not of exosome-mediated molecular transfer. Our findings therefore suggest that melanoma exosome-mediated “re-education” of the cells resulted in a novel MSC population which could be identified as MSC^PD-1+^.

### B16F1 Exosomes Augment *in vivo* Tumorigenesis and Tumor Progression

After presenting evidence for the *in vitro* tumorigenic induction potential of exosomes on cultured MSCs, we hypothesized that this phenomenon could be identified *in vivo* as well. To probe this assumption, we employed the well-known animal model, routinely used in our laboratories (46), in which tumors, developed mostly in the lungs, are induced in mice by intravenous administration of mouse B16F1 melanoma cells (to the tail vein). Then, tumor-bearing mice received buffer, or exosomes isolated from the same B16F1 melanoma cells, or exosome-induced MSC^PD-1+^ cells.

**Figure 4:**
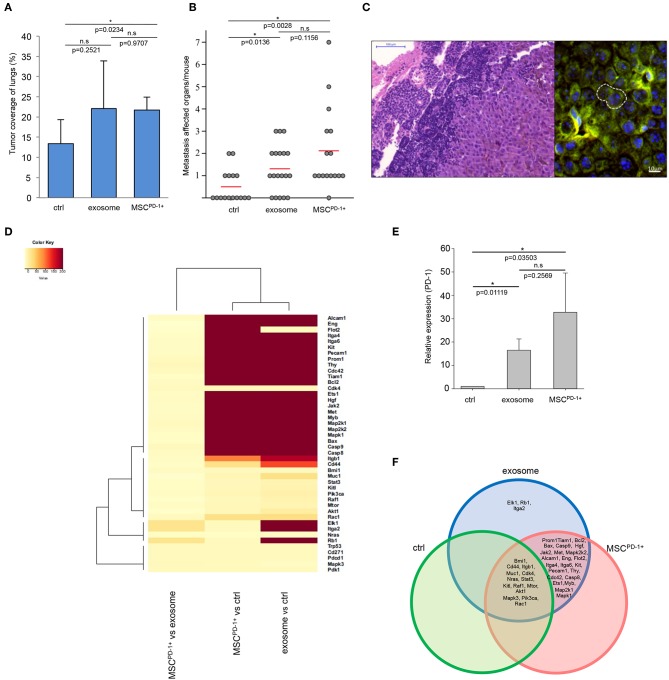
Melanoma exosomes promote tumor progression and metastasis formation *in vivo*. **(A)** Tumor coverage of lungs on day 15 in different animal groups. The graph represents mean + SD (*n* = 3). **(B)** Number of distant metastases on day 25. One dot represents one animal in each group, red lines show the average number of metastases per animal. **(C)** FISH analysis of a paraortic lymph node metastases, which showed the presence of exosome-exposed MSC. Y chromosome (red dot) of the male mouse-derived MSC was detected in the metastases of a female mouse. **(D)** Heatmap and cluster analysis of gene expression pattern, which show protooncogenic dominance in exosome or MSC^PD-1+^ groups. Robust hierarchical clustering based on fold changes in the gene expression data between selected groups divide the treated groups into several upper classes. Groups treated by exosomes or MSC^PD-1+^ were compared to untreated controls. Exosomes MSC^PD-1+^ treatments were closely related. **(E)** QRT-PCR analysis of PD-1 in the lung samples using TaqMan probes (*n* = 3). **(F)** The Venn diagram shows possible relations between a finite collections of different sets of genes measured during gene expression profiling **(D)**.

Notably, the exosome-related groups (i.e., exosome, MSC^PD-1+^) were characterized by a markedly increased size of tumor-covered lung tissues (the increase proved to be significant in the MSC^PD-1+^ group) (Figure 4A). Of further importance, we also found that in both exosome-related groups, the numbers of distant metastases were significantly elevated when compared to the control (Figure 4B), and diameter of metastases associated blood vessels significantly increased in exosome and MSC^PD-1+^ injected groups of tumor bearing mice (Figure S3). Using of cytokine and chemokine arrays, tumor supportive cytokine and chemokine levels were elevated in exosome related groups (Figure S4). As we have seen during our previous studies (46), these metastases were mostly localized to the ovaries and kidneys (and very rarely to the lymph nodes) of control tumor-bearing animals. However, besides these sites, the presence of exosomes resulted in frequent metastases in the lymph nodes and, as a new location, in the liver. Interestingly, in MSC^PD-1+^ treated mice, exosome-transformed MSCs could be identified in the para-aortic lymph nodes by FISH (Figure 4C) verifying the successful *in vivo* adherence of MSC^PD-1+^ cells.

Lung tissues of the different groups were then subjected to in-depth expressional profiling 14 days after injection of exosomes or MSC^PD-1+^ cells. Namely, QRT-PCR analysis was performed using a self-designed panel of 40 genes; besides housekeeping genes, we assessed expression of (i) proto-oncogenes; (ii) genes reportedly involved in malignant transformation; and/or (iii) genes found to be involved in melanoma development and progression.

Hierarchical cluster analysis (HCA) of the gene expression patterns clearly showed a robust proto-oncogenic dominance in lung samples of the exosome-related groups when compared to the control tissues. Indeed, we identified significant *de novo* induction of 26 and 23 genes: Alcam1, Eng, Flot2, Itga4, Itga6, Kit, Pecam1, Prom1, Thy, Cdc42, Tiam1, Bcl2, Bax, Casp9, Casp8, Ets1, Hgf, Jak2, Met, Myb, Map2k1, Map2k2, Mapk1 in both group and Elk1, Rb1, Itga2 in the exosome group. An additional six and three genes were identified with at least 10-fold increase (Cd44, Itgb1, Muc1, Pik3ca, Akt1, Rac1 in the exosome group and Cd44, Itgb1, Rac1 in the MSC^PD-1+^ group). Furthermore, six and eight genes showed at least two-fold mRNA level elevations (Bmi1, Cdk4, Stat3, Kitl, Raf1, Mtor and Bmi1, Muc1, Cdk4, Stat3, Kitl, Pik3ca, Mtor, Akt1, respectively). Figure 4D presents a heatmap of indicative data of overexpressed genes induced by exosome exposure.

Furthermore, we showed that, besides the above genes, expression of PD-1 was also significantly increased in both exosome-related groups (Figure 4E). Notably, although mRNA transcript level of PD-1 was close to two-fold in MSC^PD-1+^ lung tissues in comparison to samples in the exosome group, the difference was not significant (most probably due to the large inter-animal variability and standard error).

We constructed a Venn-diagram (Figure 4F) to show all possible logical connections between the various gene expression patterns presented in Figure 4D. Importantly, according to cluster analysis, we could not identify a single gene which was missing from the exosome-related groups in comparison to the control tumor-bearing mice. In other words, whereas these exosome-related groups did exhibit gene expression profiles which were characteristic only for them, such individual profiles could not be detected in the control group.

Specifically, the following gene expression patterns were defined:

- three genes (Elk1, Rb1, Igta2) were exclusively induced only in the exosome-treated group;
- twenty-three genes (Prom1, Tiam1, Bcl2, Bax, Casp9, Hgf, Jak2, Met, Mapk2k2, Alcam1, Eng, Flot2, Itga4, Itga6, Kit, Pecam1, Thy, Cdc42, Casp8, Ets1, Myb, Map2k1, Mapk1) were found to be upregulated both in the exosome and MSC^PD-1+^ cell treated groups;
- fourteen genes (Bmi1, Cd44, Itgb1, Muc1, Cdk4, Nras, Sat3, Kitl, Raf1, Mtor, Akt1, Mapk3, Pik3ca, Rac1) were found to be upregulated in all three groups.

Finally, it should be noted that the dramatic gene expression alterations seen in the exosome-related groups were exclusively due to the presence of the exosomes as the “MSC^PD-1+^ only” cluster contained no genes (Figure 4F).

### Melanoma-Derived Exosomes Promote Tumorigenic and Cell Survival Signaling Pathway(s)

**Figure 5:**
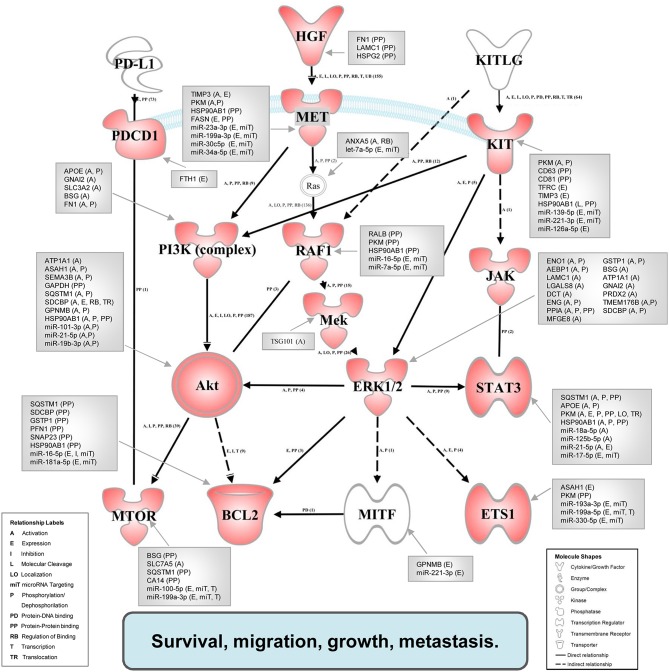
Integrated associative network of the *in vivo* overexpressed genes supplemented with the interacting exosomal factors. The network of overexpressed genes (red symbols) was conceived by us based on literature data. The relationships between molecules were supported also by the IPA knowledge base. Network visualization was performed using the Path Explorer tool of the IPA Path Designer. The exosomal proteins and miRNAs (gray boxes) were connected to elements of the network by the Grow tool of the IPA Path Designer based on experimental data of the IPA knowledge base. Activation of the established network by exosomal components may support the survival, migration, growth, and metastasis of tumor cells.

Figure 5 shows the map of interaction pathways based on overexpressed molecules (red symbols) in *in vivo* experiments. We detected overexpressed elements of three main pathways which do participate in tumor progression and metastasis formation (47). Using the IPA Path Designer Grow tool, we generated an interaction map which contains proteins (or their established complexes) encoded by the overexpressed genes, and exosomal miRNAs and proteins (gray boxes) which were previously shown to control or affect the marked signaling molecules.

To demonstrate these results and to explore the potential causative exosomal factors, we again performed an IPA. The analysis allowed further refinement of the underlying molecular processes and pathways involved in the summarized effect of this gene expression profile. The previously described 40-gene QRT-PCR panel was enriched in genes related to cellular movement and migration, cell survival and connective tissue development and function, involving tumor cells. Additional processes included growth and proliferation of tumor cells as well as the PD-1:PD-L1 interaction (and its consequences). Focusing on PD-1 and mTOR pathway, a network was built from the overexpressed genes, and their complexes known to be related to tumor progression using the Path Explorer tool in the IPA Path Designer. Then a list was generated that contained the exosomal proteins detected by MS and the exosomal miRNAs identified by SOLiD sequencing. The Grow tool in the IPA Path Designer revealed significant interactions between the exosome-induced expression network and the molecular content of these vesicles. The resulting interaction network shows both direct and indirect interactions, but it contains only experimentally observed relationships based on the Ingenuity Knowledge Base. This network demonstrates that 61 exosomal molecules (Table 4) may affect tumor progression through pathways controlled by key components including MET, Ras, RAF1, Mek, ERK1/2, MITF, BCL2, PI3K, Akt, mTOR, PD-1, KIT, JAK STAT3, or ETS1.

**Table 4:** List of exosomal proteins and miRNAs connected to the integrated associative network by IPA Path Designer.

**ID**	**Symbol**	**Entrez gene name**	**Symbol**	**Seed regio**	**ID**
Q640N1	AEBP1	AE binding protein 1	let-7a-3p	UAUACAA	mmu-let-7a-1-3p, mmu-let-7b-3p
P48036	ANXA5	Annexin A5			mmu-let-7c-2-3p, mmu-let-7f-1-3p
P08226	APOE	Apolipoprotein E	let-7a-5p	GAGGUAG	mmu-let-7a-5p, mmu-let-7b-5p
Q3TWT5	ASAH1	N-acylsphingosine amidohydrolase 1			mmu-let-7c-5p, mmu-let-7d-5p
Q3TXF9	ATP1A1	ATPase Na^+^/K^+^ transporting subunit alpha 1			mmu-let-7e-5p, mmu-let-7f-5p
O55107	BSG	Basigin (Ok blood group)			mmu-let-7g-5p, mmu-mir-98-5p
Q9WVT6	CA14	Carbonic anhydrase 14	miR-100-5p	ACCCGUA	mmu-mir-99a-5p, mmu-mir-99b-5p
P41731	CD63	CD63 molecule	miR-125b-5p	CCCUGAG	mmu-mir-125a-5p, mmu-mir-125b-5p
P35762	CD81	CD81 molecule			mmu-mir-351-5p
P29812	DCT	Dopachrome tautomerase	miR-126a-5p	AUUAUUA	mmu-mir-126a-5p
Q3UAM9	ENG	Endoglin	miR-139-5p	CUACAGU	mmu-mir-139-5p
P17182	ENO1	Enolase 1	miR-16-5p	AGCAGCA	mmu-mir-15a-5p, mmu-mir-15b-5p
P19096	FASN	Fatty acid synthase			mmu-mir-16-5p, mmu-mir-195a-5p
P11276	FN1	Fibronectin 1			mmu-mir-322-5p, mmu-mir-497a-5p
P09528	FTH1	Ferritin heavy chain 1	miR-17-5p	AAAGUGC	mmu-mir-106b-5p, mmu-mir-17-5p
P16858	GAPDH	glyceraldehyde-3-phosphate dehydrogenase			mmu-mir-20a-5p, mmu-mir-93-5p
P08752	GNAI2	G protein subunit alpha i2	miR-181a-5p	ACAUUCA	mmu-mir-181a-5p, mmu-mir-181b-5p
Q3TAV1	GPNMB	Glycoprotein nmb			mmu-mir-181c-5p, mmu-mir-181d-5p
P19157	GSTP1	glutathione S-transferase pi 1	miR-18a-5p	AAGGUGC	mmu-mir-18a-5p
P11499	HSP90AB1	Heat shock protein 90 alpha family class B member 1	miR-193a-3p	ACUGGCC	mmu-mir-193a-3p
B1B0C7	HSPG2	Heparan sulfate proteoglycan 2	miR-199a-3p	CAGUAGU	mmu-mir-199a-3p, mmu-mir-199b-3p
P02468	LAMC1	Laminin subunit gamma 1	miR-199a-5p	CCAGUGU	mmu-mir-199a-5p, mmu-mir-199b-5p
Q3U2W5	LGALS8	Galectin 8	miR-19b-3p	GUGCAAA	mmu-mir-19a-3p
P21956	MFGE8	Milk fat globule-EGF factor 8 protein			mmu-mir-19b-3p
P62962	PFN1	Profilin 1	miR-21-5p	AGCUUAU	mmu-mir-21a-5p
P52480	PKM	Pyruvate kinase, muscle	miR-221-3p	GCUACAU	mmu-mir-222-3p
P17742	PPIA	Peptidylprolyl isomerase A	miR-223-3p	GUCAGUU	mmu-mir-223-3p
Q61171	PRDX2	Peroxiredoxin 2	miR-23a-3p	UCACAUU	mmu-mir-23a-3p
Q8CCG5	RALB	RAS like proto-oncogene B			mmu-mir-23b-3p
O08992	SDCBP	Syndecan binding protein	miR-30c-5p	GUAAACA	mmu-mir-30a-5p
Q0VGP2	SEMA3B	Semaphorin 3B			mmu-mir-30b-5p
P10852	SLC3A2	Solute carrier family 3 member 2			mmu-mir-30c-5p
Q3UQM7	SLC7A5	Solute carrier family 7 member 5			mmu-mir-30d-5p
O09044	SNAP23	Synaptosome associated protein 23			mmu-mir-30e-5p
Q64337	SQSTM1	Sequestosome 1	miR-330-5p	CUCUGGG	mmu-mir-326-3p
Q542D9	TFRC	Transferrin receptor	miR-34a-5p	GGCAGUG	mmu-mir-34a-5p
P39876	TIMP3	TIMP metallopeptidase inhibitor 3			mmu-mir-34b-5p
Q9R1Q6	TMEM176B	Transmembrane protein 176B			mmu-mir-34c-5p
Q3UCW0	TSG101	Tumor susceptibility 101	miR-7a-5p	GGAAGAC	mmu-mir-7a-5p

Taken together, these findings demonstrate that interaction between exosomes and MSCs induces a tumor-like phenotype with PD-1 overexpression of naïve MSC *in vitro* and a fast tumor progression *in vivo*.

## Discussion

Considering the fact that metastatic complications are responsible for almost 90% of cancer-driven mortality (48), a deep understanding of tumor metastatic processes is one of the most important challenges in both fundamental and applied research directions in tumor biology. Whilst certain organotropic aspects of tumor cells were addressed by Paget's “seed-and-soil” hypothesis in the early nineteenth century (49), the detailed description of the spatiotemporal characteristics of the metastatic processes requires an extended model. MSCs, as multipotent stromal cells, are important players in dynamic tumor microenvironment (12). MSCs could stimulate angiogenesis via paracrine signaling and participate in metastasis formation to promote the epithelial-mesenchymal transition (12). Consequently, in the last decade, numerous studies suggested the importance of soluble and vesicle-like components of the tumor microenvironment in epithelial-mesenchymal transition as crucial factors in the metastatic establishment (50). Among these, recent findings of intercellular communication mechanisms unquestionably highlighted the crucial role of EVs (e.g., exosomes) serving as “information packages” within the coordinated cell-to-cell signaling (51).

In the current study, we demonstrate that MSCs, widely abundant in solid tumors as well as in healthy tissues, undergo a marked re-education process upon communication with metastatic cancer cells via exosome-mediated information transfer. This transformation process results in characteristic response-patterns corresponding to a given cancerous cell lines (12).

First, we intended to precisely define the EV population which we later employed via *in vitro* and *in vivo* experimental approaches. Besides applying the experimental requirements of the International Society for Extracellular Vesicles (ISEV) (i.e., determination of shape, size distribution and protein markers) (52), we thoroughly defined the miRNA and protein content of melanoma exosomes. These systematic assays concluded that the isolated vesicles contained factors, which may regulate signaling pathways related to cell death and survival, cellular movement, development and cell proliferation. Given the appropriate uptake of these vesicles by the targeted, multipotent recipient cells, it is highly probable that most signaling pathways mentioned above were significantly affected. In accordance with previous results in the literature, we showed successful internalization of exosomes by recipient MSCs. As expected, following 72 h of exposure, MSCs displayed accelerated proliferation presumably initiated by the transforming exosome cargo. Moreover, melanoma exosomes were able to hinder the induction of the cell death response commonly triggered by TNFα. TNFα is a frequently identified, soluble, tumor-suppressing factor in cancerous microenvironments (53). This inhibition strongly suggested that exosomes initialize and sustain malignant transformation of MSCs.

Further, we described numerous, exosome-induced phenotypic manifestation of the above malignant re-education process in the recipient cells. Apart from identifying tumor markers, the transformation of exposed cells is characterized by markedly modified gene expression patterns. As discussed previously, specific gene expression alterations are often associated with specific steps of cancerous transformation. Malignant cell lines possess altered expression of genes responsible for apoptotic regulation (Bax, Bcl2, caspases), regulating metastasis (integrins, ITGA2-6, ITGB1, KIT) or various growth factors (ETS1, HGF, MET, etc.). Importantly, we successfully identified two melanoma-specific markers, i.e., MITF (Microphthalmia-associated transcription factor) and MLANA (Melanoma antigen recognized by T-cells) in the exosome-exposed MSCs. MLANA and its regulator MITF play a fundamental role in melanocyte development, tumor progression and are overexpressed in melanoma cells (54). The presence of these markers in exosome-exposed MSCs shows effective transfer of the encoded tumorigenic information, and may have a pivotal role in disease progression. Besides, we also identified a characteristic set of genes, belonging mostly to proto-oncogenes, whose expression was significantly elevated upon exosome treatment. Among these molecules, we emphasize the increased expression of CD44 which was previously assigned as cancer stem cell marker and of mTOR, a characteristic indicator of the engagement of the PD-1:PD-L1 pathway (15). Indeed, activation of mTOR signaling is particularly important. As it was previously demonstrated by Kleffel et al., overexpression of PD-1 in exceptionally aggressive melanoma subpopulations facilitated tumor progression by activating the mTOR signaling pathway (actually, this response was highly unexpected compared to that of the classic PD-1:PD-L1 dependent T-cell anergy, a crucial aspect of immunotherapy in the clinics) (15). Quite strikingly, in exosome treated MSCs, we detected a significant induction of PD-1 and mTOR. These novel data strongly support that these vesicles might trigger the formation of an aggressive, melanoma-like subpopulation of re-educated recipient MSCs.

Our findings therefore propose that the re-educated melanoma-like MSC^PD-1+^ subpopulation, originating from multipotent tissue-derived MSCs, is an autonomous entity. It is characterized by typical properties of cancerous transformation such as hyperproliferation, resistance to apoptosis, expression of melanoma markers, proto-oncogenic gene expression patterns, and PD-1 over-expression. Indeed, those melanoma exosomes that induced *in vitro* generation of the melanoma-like MSC^PD-1+^ cells, also facilitated *in vivo* metastasis formation in the lungs or other distant organs of treated animals. Actually, our findings that melanoma exosomes or exosome re-educated MSC^PD-1+^ promote tumor progression, are in good accordance with previous finding of Kleffel et al. describing that shRNA or antibody mediated inhibition of PD-1 signaling inhibits metastasis formation and tumor progression in experimental animals (15). Assessment of the expression patterns of genes related to tumor progression in the exposed animal tissues revealed numerous cases of exosome-induced overexpression; this supports our hypothesis that melanoma exosomes should be considered as oncosomes (6).

Our results also demonstrated that melanoma exosomes also generated a characteristic signaling pattern in MSC^PD-1+^ recipient population. As it was shown in numerous studies PI3K/Akt, Ras/MAPK, and STAT3 pathways are commonly activated in tumor cells (47, 55). In this study, based on our *in vitro* and *in vivo* results and literature data, we introduce a novel, comprehensive network of common tumor-related proteins, such as PD-1, MET, RAF1, STAT3, BCL2, or mTOR. The network highlights not only the relationship of these elements, but also contains upstream exosomal regulating factors which may contribute to the activation of tumorigenic signaling, and hence, fast tumor progression. According to our best knowledge, this associative network between overexpressed genes and the potential exosomal inducers, is the first tumor progression signaling pattern which connected experimental response-patterns with experimentally detected exosomal-molecular-patterns. Further, our data also indicate that the above complexity of exosomal communication requires system-level approaches.

Finally, it is important to note that our conclusions are not based on *in silico* predictions exclusively, but rather on carefully designed and systematically executed *in vitro* and *in vivo* experiments, all suggesting that the specific, robust, molecular content of the isolated exosomes can indeed generate a unique intercellular niche responsible for the re-education of neighboring cells via oncogenic transformation. These re-educated melanoma-like MSC^PD-1+^ cells, in turn, facilitate metastatic disease progression via ignition of a complex series of subsequent events both locally and systematically. Thus, based on our results and recently published, additional evidences about exosomes, there is an urgent demand to supplement and extend the outdated “seed-and-soil” hypothesis with the oncosome-driven re-education process.

## Data Availability Statement

All datasets generated for this study are included in the manuscript/Supplementary Files.

## Ethics Statement

All animal experiments were performed in accordance with national (1998. XXVIII; 40/2013) and European (2010/63/EU) animal ethics guidelines. The experimental protocols were approved by the Animal Experimentation and Ethics Committee of the Biological Research Centre of the Hungarian Academy of Sciences and the Hungarian National Animal Experimentation and Ethics Board (clearance number: XVI./03521/2011 and XVI./78/2018).

## Author Contributions

EG-S: acquisition, analysis and interpretation of data (*in vitro* and *in vivo* experiments), drafting or revising the article. MH and GD: acquisition, analysis and interpretation of data, drafting or revising the article. IBN: histology. JM: acquisition, analysis and interpretation of data-*in vivo* QRT-PCR. ÁZ: acquisition, analysis and interpretation of data (MLANA, MITF). ÉH-G: acquisition, analysis and interpretation of Proteomics data. RK: providing and supervising of stem cell protocols. IN: acquisition, analysis and interpretation of miRNA data. PH: supervising of machine learning. ÁB: high content screening. ÁS: acquisition, analysis and interpretation of machine learning data. MK: acquisition, analysis and interpretation of confocal data. TP: drafting or revising the article, PD-1 QRT-PCR. BB: acquisition, analysis of data (*in vitro* QRT-PCR). ME: STORM microscopy. ZS: AFM microscopy. ZV: acquisition, analysis and interpretation of apoptosis data. EB: drafting or revising the article. LK: clinical relevance and drafting or revising the article. TB: conception and design and drafting or revising the article. KB: conception and design, acquisition of data, analysis and interpretation of data, drafting or revising the article and founding acquisition.

### Conflict of Interest

The authors declare that the research was conducted in the absence of any commercial or financial relationships that could be construed as a potential conflict of interest.
