# Supplementary material for: Melanoma-Derived Exosomes Induce PD-1 Overexpression and Tumor Progression via Mesenchymal Stem Cell Oncogenic Reprogramming
Source: Front Immunol. 2019 Oct 18;10:2459. doi: 10.3389/fimmu.2019.02459 (PMC6813737; doi:10.3389/fimmu.2019.02459)
Supplement: Supplementary file 1 [file Data_Sheet_1.pdf]

Supplementary Material

Melanoma-Derived Exosomes Induce PD-1 Overexpression and Tumor Progression via Mesenchymal Stem Cell Oncogenic Reprogramming

Edina Gyukity-Sebestyén, Mária Harmati, Gabriella Dobra, István B Németh, Johanna Mihály, Ágnes Zvara, Éva Hunyadi-Gulyás, Róbert Katona, István Nagy, Péter Horváth, Árpád Bálind, Ábel Szkalisity, Mária Kovács, Tibor Pankotai, Barbara Borsos, Miklós Erdélyi, Zsolt Szegletes, Zoltán J Veréb, Edit I Buzás, Lajos Kemény, Tamás Bíró, Krisztina Buzás

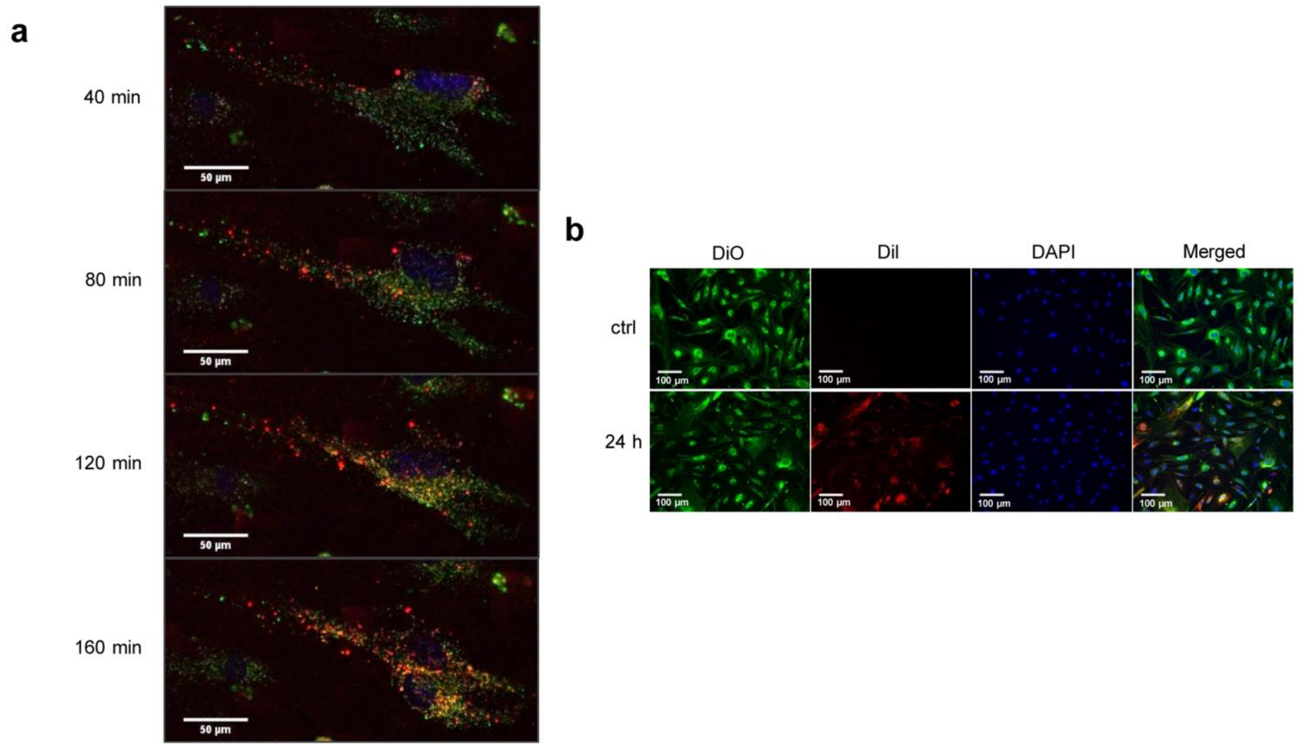

Figure S1. Kinetics of exosome uptake by MSCs. (**a**) Fluorescent images of exosome uptake by MSCs in early timepoints. DiO (green fluorescent lipid dye) and Hoechst 33342 (nucleic acid stain)-labeled cells were exposed to Dil (red fluorescent lipid dye)-labeled exosomes and were investigated by a Celldiscoverer 7 automated live cell imaging system (Zeiss). Internalization of exosomes was visible as early as 40 min after exosome exposure. (**b**) Fluorescent images of exosome uptake by MSCs 24 h after exosome exposure. Images were taken by an Operetta high content screening system. The amount of internalized exosomes showed cell to cell variation.

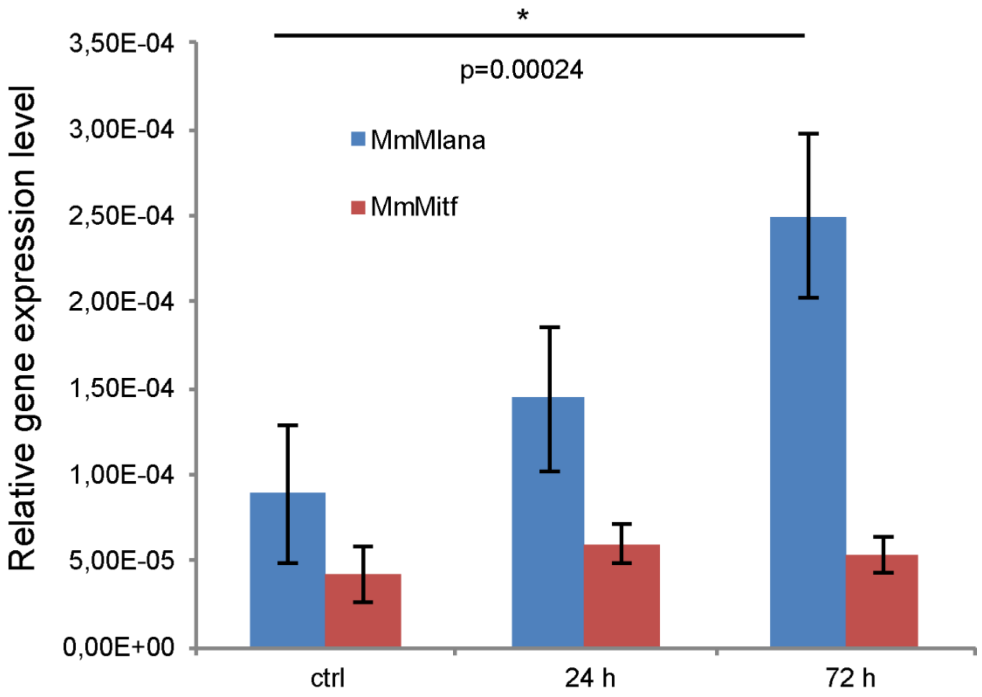

Figure S2. QRT-PCR analysis of Mlana and Mitf in exosome-exposed MSCs. The Mlana and Mitf mRNA levels were determined by QRT-PCR using a SyberGreen protocol after 24 h and 72 h of exosome exposure. Results are presented as mean ± SD (n=3).

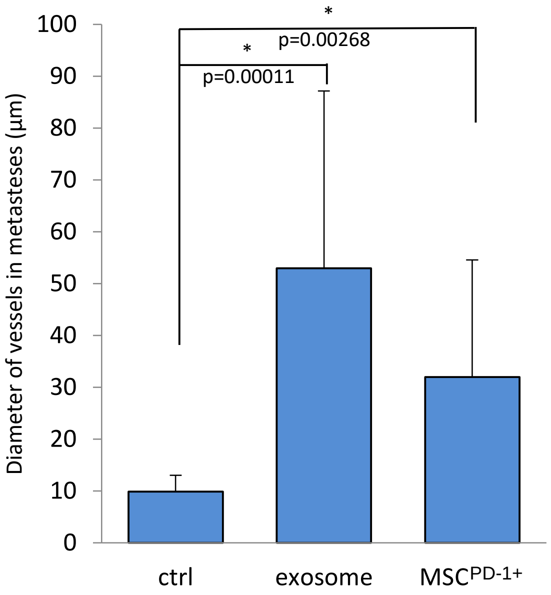

Figure S3. Quantification of vessel diameter of tumor-associated blood vessels in melanoma metastases. Diameter of vessels significantly increased in the metastases of exosome and MSC^{PD-1+} injected groups of tumor-bearing mice inside the lung metastases.

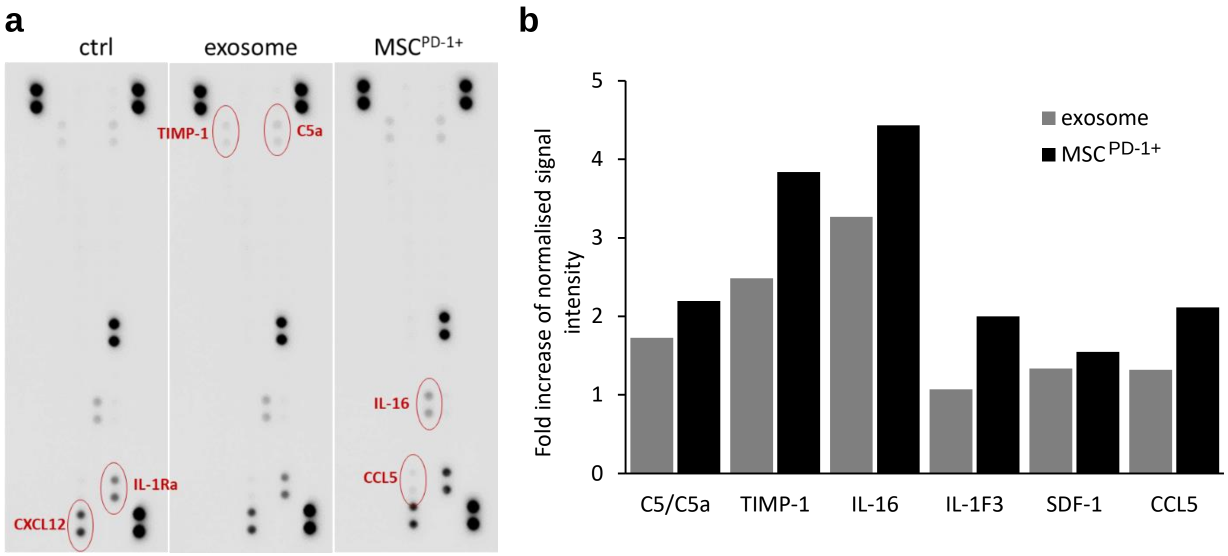

Figure S4. Exosomes and MSC$^{PD\text{-}1+}$ administration-induced cytokine profiles in the lung tissues of tumor-bearing mice. (a) cytokine array panels of the lung tissue lysates, cytokines with elevated levels are marked. (b) bar graph shows the fold increase values of cytokines for both groups compared to the ctrl one. Data were obtained by densitometry.

Table S1. Mlana and Mitf primers used in QRT-PCR

Gene name	Symbol	Ref. seq. ID	Forward primer sequence	Reverse primer sequence
**melan-A**	Mlana	NM_029993.1	ggtcctggggattgctct	caatatgacgccttttgtcca
**microphthalmia-associated transcription factor**	Mitf	NM_008601.3	ctaagtggtctgcggtgtctc	ggttttccaggtgggtctg

Primers were designed using the online Roche Universal Probe Library Assay Design Center. The quality of the primers was verified by MS analysis provided by Bioneer (Daejeon, Korea).

Table S2. Treatment schedule of animals

Group	B16F1	Control buffer	$MSC^{PD\text{-}1+}$ i.v.	B16F1 exosome i.v.
ctrl	+	+	-	-
$MSC^{PD\text{-}1+}$	+	-	+	-
exosome	+	-	-	+
